# The impact of changes to work circumstances enforced by COVID-19 on anxiety: a systematic review

**DOI:** 10.1186/s13643-025-02950-9

**Published:** 2025-10-15

**Authors:** Stefania D’Angelo, Elena Zaballa, Georgia Ntani, Ilse Bloom, Karen Walker-Bone

**Affiliations:** 1 MRC Lifecourse Epidemiology Centre, University of Southampton, Southampton, UK; 2 MRC Versus Arthritis Centre for Musculoskeletal Health and Work, MRC Lifecourse Epidemiology Centre, University of Southampton, Southampton, UK; 3 NIHR Southampton Biomedical Research Centre, University of Southampton and University Hospital Southampton NHS Foundation Trust, Southampton, UK; 4 School of Public Health and Preventive Medicine, Monash University, Melbourne, VIC 3004 Australia

**Keywords:** Systematic review, Changes in employment, COVID-19 pandemic, Older workers, Anxiety

## Abstract

**Background:**

The COVID-19 pandemic enforced changes on employment circumstances for all workers but older workers experiencing job loss are less likely to return to work than younger individuals. Under normal circumstances, job loss is a well-recognised risk factor for poor mental health, while it is unclear whether working from home is beneficial or harmful to mental health.

We systematically reviewed the literature to explore the association between enforced changes in employment (job loss, working from home or being furloughed) and anxiety in the adult population, with a particular focus on older workers.

**Methods:**

The protocol was registered in June 2021 in the International Prospective Register of Systematic Reviews database. We searched Medline, Embase, PsycInfo and CINAHL (January 2020–July 2023) databases for studies including older adults (some of the study sample were workers aged over 50 years). Results were presented by narrative review, complemented by a vote-counting technique and effect direction plots to summarise the relationship between exposures and anxiety.

**Results:**

Forty-eight studies from several countries met the inclusion criteria, including 39 cross-sectional and nine longitudinal studies. The prevalence of anxiety varied between studies due to different tools and cut-offs chosen, reaching as high as 63% in one study. The vote-counting method showed convincing evidence that job loss since lockdown negatively impacted anxiety overall and among people aged 50 and over. Inconsistent results were observed across studies investigating the effect of working from home or furlough on anxiety.

**Conclusion:**

Disruption of employment during the pandemic and related lockdowns has increased anxiety levels in the adult population and among older workers. More research is needed to know how persistent these effects are and to identify strategies to support those most affected.

**Systematic review registration:**

The protocol of the systematic review was registered in June 2021 in the International Prospective Register of Systematic Reviews database (PROSPERO: CRD42021260499), and it is provided as supporting information (Additional File 1).

**Supplementary Information:**

The online version contains supplementary material available at 10.1186/s13643-025-02950-9.

## Background

Western countries, including the UK, are experiencing a demographic shift towards an ageing population. In response, governments are implementing policies aimed at extending working lives and encouraging individuals to remain in paid employment to older ages [1, 2]. These measures include increasing the statutory retirement age, offering financial incentives to delay retirement, promoting flexible working arrangements, and investing in lifelong learning and reskilling initiatives [3, 4].

Since the onset of the COVID-19 pandemic in 2020, many workers have experienced disrupted employment: many were forced out of employment prematurely, while others experienced a sudden shift to working from home [5]. According to the International Labour Organization, in April 2020 a sizeable 68% of the global workforce were in countries where workplace closures were either recommended or mandated [6]. In the UK alone, in the period between January–March 2020 and November–January 2021, the employment rate went from 76.3% down to 74.6% and unemployment levels rose throughout 2020 [7]. Previous studies have shown that unemployment [8] and involuntary transitioning from paid work to unemployment [9] are important risk factors for poor mental health among any age workers, and older workers [10]. The mechanisms behind this association were addressed in the latent deprivation model proposed by Jahoda [11], in which the lack of five latent functions of employment (time structure, social contact, collective purpose, status, and activity) impacted negatively on mental health. We therefore anticipated that people with imposed job loss because of the pandemic were at high risk of experiencing a worsening in their mental health. An unexpected job loss, especially among older workers, is of particular concern as it could significantly compromise their prospect of working to older ages. This is due to their difficulty re-entering the workforce once they experience involuntary job loss [12]. In addition, people aged 50 and older are more likely than younger individuals to live with multi-morbidities [13]. The COVID-19 pandemic could therefore have important long-term negative health consequences for this group, potentially increasing the prevalence of co-morbidities per person.

It is unclear whether working from home is beneficial or detrimental for mental health, especially when this work pattern has been rapidly imposed rather than planned and implemented as part of a common agreement between the employee and employer. A review [14] comprising studies published up to 2020, reported inconclusive findings about the relationship between working from home and mental health. Some of the studies included in the review identified working from home to be associated with increased stress [15, 16], fatigue [17], depression [18] or mental exhaustion [19]. Other studies highlighted a positive impact of working from home such as better quality of life [20, 21], and improved wellbeing [22, 23]. The review authors pointed out that adequate organisational support and formal co-worker and technical support are paramount for working from home to be healthy [14]. However, this evidence pre-dates the pandemic and may not be relevant to working from home imposed by the pandemic.

There is also limited evidence post-pandemic about the impact of being temporarily out of work, paid at 80% of normal salary, but still retaining a job to return to (i.e. furlough) on mental health. Some studies found furlough to increase the risk of depression [24], or more generally worsen mental health [25], as significantly as job loss. Others, however, suggested that retaining a job, despite being furloughed, benefitted mental health [26].

Studies conducted pre-pandemic have demonstrated that immediate stress reactions following a traumatic incident—like the COVID-19 pandemic—can result in negative mental health consequences including depression and anxiety [27]. The literature reviewed above suggests that changes in employment because of the pandemic may add to the anxiety generated by a pandemic and exacerbate these effects.

Therefore, the objective of this systematic review was to critically appraise the body of published evidence evaluating the impact of enforced changes in employment during the COVID-19 pandemic on mental health of the adult population, with a focus on workers aged 50 and older. As a measure of mental health, we chose anxiety, as one of the most common mental health conditions. This was a pragmatic decision, as exploring several measures of mental ill-health would not have been manageable.

## Material and methods

The protocol of the systematic review was registered in June 2021 in the International Prospective Register of Systematic Reviews database (PROSPERO: CRD42021260499), and it is provided as supporting information (Additional File 1). Following the Population, Intervention, Comparison and Outcome (PICO) format, our research question was as follows: what have been the effects of enforced changes in employment circumstances that occurred since the COVID-19 pandemic on levels of anxiety in the adult population?

### Search strategy

The study followed The Preferred Reporting Items for Systematic Reviews and Meta-analysis (PRISMA) guidelines 2020 and the PRISMA checklist which can be found in the supplementary material (Additional File 2). Search for the systematic review was performed in four electronic databases: MEDLINE and Embase (Ovid platform), PsycInfo, and CINAHL (EBSCO platform). Search strategies are available as Supporting material (Additional File 3). An initial search included papers published from January 2020 to May 2022, and this was updated on the 22nd of July 2023. The search was restricted to papers published in either English or Italian to align with the language proficiency of the study team. Only peer-reviewed papers were included. Returns from searches of all four databases were imported into EndNote and duplicates were identified and removed. Conference abstracts, editorials, notes and letters were excluded. A snowball search was conducted using the Web of Science database, and the reference list of selected manuscripts to identify potentially relevant papers that might have been missed with the initial database search.

### Inclusion and exclusion criteria

We included observational studies of all types if (i) they involved the adult population (≥ 18 years of age), provided that the sample studied included people aged ≥ 50 and (ii) they investigated the effect of changes in employment (job loss, working from home, change in working hours, furlough) since the COVID-19 pandemic on anxiety. We excluded studies when employment change was unrelated to the COVID-19 pandemic. We included papers that evaluated anxiety as the outcome, measured with any validated or not validated tool. Manuscripts only providing descriptive statistics and not associations between variables were included, provided they compared anxiety across categories or levels of changes in employment. These are named “descriptive studies” throughout, as opposed to “analytical studies”. Additionally, papers exploring the research question in relation to a specific occupational group/s or on a sample with a specific health condition, were excluded as their findings would not be applicable to the general population.

### Screening

**Fig. 1:**
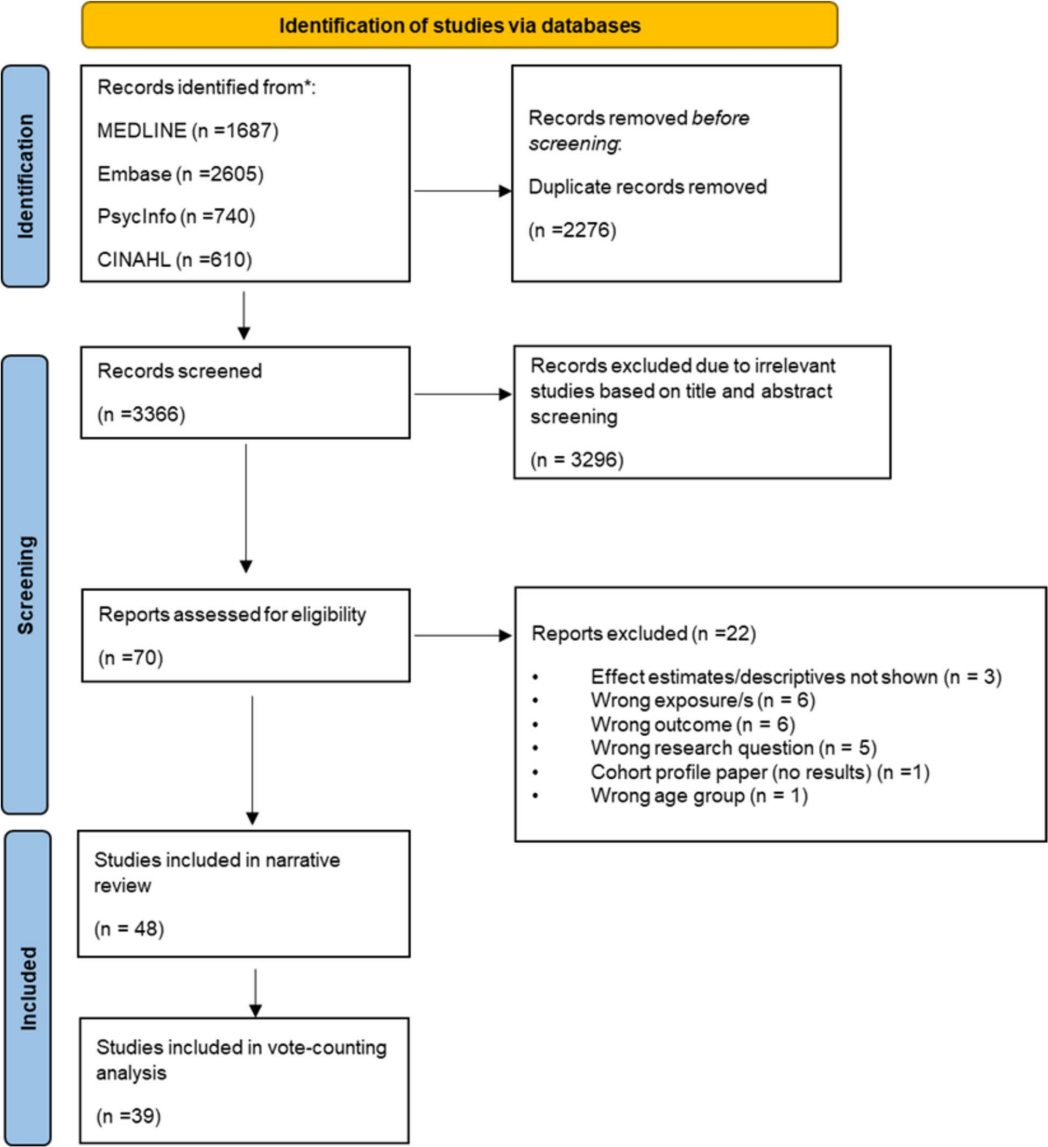
PRISMA flow-chart

Titles and abstracts were initially screened for eligibility by one reviewer (SD), who classified papers as “to include”, “to exclude” or “uncertain”. A second reviewer (EZ) screened all those that were categorised as “uncertain”, “to include”, and an additional 10% of those classified as “to exclude”. After discussing discrepancies, both reviewers (SD and EZ) agreed on which studies required to be full-text screened to identify those that were suitable for inclusion. Reasons for exclusion are detailed in the flow-chart (Fig. 1) [28].

### Data extraction

A data extraction form was created with all fields relevant to this review. A draft version was trialled on a sample of studies by SD and KWB. Two reviewers, SD and EZ extracted information from all included papers independently and compared forms afterwards. Data extracted included: year and first author of the article, title, country, data collection period, study design, check for eligibility criteria, sample size overall and stratified by sex and for the age group 50 and older (if available), age of sample, response rate (if specified), definition and prevalence of the exposure/s and of the outcome, details of whether the study was purely descriptive or it provided estimates of associations, statistical methods used, confounders considered and risk estimates overall and for the age group 50+ (if available).

### Collecting unpublished material

In line with recommendations in the Cochrane handbook [29], and due to our special focus on older workers (≥ 50 years), we contacted all corresponding authors to ask for additional information regarding any unpublished analyses they were willing to provide, based on the age group 50+.

### Risk of bias assessment

Quality of included studies was assessed using a risk of bias tool, based on a combination of the Scottish Intercollegiate Guidelines Network (SIGN) template for cohort studies [30] and the Joanna Briggs Institute (JBI) checklist for cross-sectional studies [31] and is provided as Supplementary material (Additional File 5). The risk of bias tool was developed and trialled by KWB and SD. The risk of bias checks were carried out independently by SD and KWB, and possible outcomes were as follows: “unacceptable”, “acceptable”, “medium quality”, “high quality”. Results were compared, and discrepancies were discussed to reach an agreement.

### Evidence synthesis

Findings were synthesised narratively according to type of exposure and study design. The initial aim was to synthesise findings on people aged 50 and older using meta-analysis; however, due to the heterogeneity of exposures and tools used to measure anxiety, this was not possible. Therefore, to provide a quantitative summary of the findings, we employed a “vote-counting” technique. Following the methodology from the Cochrane handbook [32], we first categorised each effect estimate as either showing benefit or harm to health. In this case, a beneficial effect on health corresponds to a decrease in anxiety. A sign test was then performed to test the null hypothesis of an equal number of positive and negative results. To perform the sign test, we counted the number of effects showing benefit and those showing harm for each exposure analysed. Inconclusive results were excluded from the calculation. Neither statistical significance nor the size of the effect was considered in the computation. An effect direction plot was then created for each exposure of interest. This plot uses arrows to visually display the direction of effect of the association within each study [33]. The process was conducted separately by type of study design.

## Results

### Study characteristics

The PRISMA flow chart showing the studies included in this review is available in Fig. 1. The initial search retrieved 5642 papers. After removing duplicates, 3366 titles and abstracts were screened. This process yielded 70 papers as potentially relevant. Assessment of full text further excluded 22 papers for the following reasons: effect estimates/descriptives were not reported (*n* = 3): wrong exposure (*n* = 6); wrong outcome (*n* = 6); wrong research question (*n* = 5); cohort profile paper (*n* = 1), wrong age group (*n* = 1). A snowball search did not retrieve any additional papers. Therefore, 48 papers were eligible to be included.

A summary of the main characteristics of the 48 articles included, along with the quality/risk of bias assessment is shown in Table 1. Studies are presented by design: the majority had a cross-sectional design (*n* = 39), while the remaining nine had a longitudinal design. Studies’ locations varied widely, although the majority were conducted in high-income countries such as the USA and Australia. Data were collected at different phases of the pandemic although most studies focused on the initial phases of lockdown, in 2020. The number of study participants ranged from a minimum of 186 [64] to a maximum of 1,576,770 [80]. The breakdown of participants by age was not always reported, with only 34 of the 48 studies showing the proportion of middle-aged participants in their sample (the threshold to define middle-aged varied from 50 to 55 and over depending on the study). In most studies, women were more represented than men. A total of nine of the 48 studies were purely descriptive, while 39 showed at least one association between the exposure/s and anxiety.

**Table 1:** Characteristics of studies included in the systematic review, by type of study design

Author and Year	Country	Data collection period	Number of participants	Gender breakdown, *N* (%)	Participants aged 50+, *N* (%)	Overall quality
Cross-sectional studies						
Abdalla *et al, *2021 [34]	USA	31st March–13th April 2020	1450	Men = 725 (48.2%) Women = 725 (51.8%)	Age 60+ = 366 (29.9%)	Acceptable (+)
Abrams* et al, *2021 [35]	USA	2nd April–31st May 2020	6264	Not reported	All aged 55+	Medium quality (++)
Alsaif* et al,* 2022 [36]	Saudi Arabia	Not specified	754	Men = 408 (54.1%) Women = 346 (45.9%)	Age 56–65 = 27 (3.6%)	Acceptable (+)
Amer* et al,* 2022 [37]	Saudi Arabia	May 2020–June 2020	858	Men = 368 (42.9%) Women = 489 (57.1%)	Age 50–65 = 105 (12.3%) Age 65+=3 (0.4%)	Acceptable (+)
Burhamah* et al,* 2020 [38]	Kuwait	25th–30th May 2020	4132	Men = 1268 (30.7%) Women = 2864 (69.3%)	Age 51+ = 1241 (30.0%)	Acceptable (+)
Burstyn* et al,* 2021 [39]	Philadelphia, USA	17th April–3rd July 2020	911	Not reported	Men Age 55+ = 81 (34.6%) Women Age 55+ = 197 (29.7%)	Medium quality (++)
Dawel* et al,* 2020 [40]	Australia	28th–31st March 2020	1296	Men = 645 (49.8%) Women = 649 (50.2%) 2 missing values	Age 50+ = 549 (42.4%)	Medium quality (++)
De Miquel* et al,* 2022 [41]	Spain	June 2020	2381	Prevalence (95%CI) Men = 47.48% (45.39–49.58%) Women = 52.53% (50.42–54.61%)	Not reported	Acceptable (+)
Elezi* et al,* 2020 [42]	Albania	4th April–29th April 2020	1678	Men = 449 (26.8%) Women = 1,229 (73.2%)	Age 46–55 = 68 (4.1%)	Acceptable (+)
Fiorenzato* et al,* 2021 [43]	Italy	29th April–17th May 2020	1215	Men = 351 (28.9%) Women = 864 (71.1%)	Age 45–65 = 429 (35.3%)	Acceptable (+)
Fisher* et al,* 2020 [44]	Australia	3rd April–3rd May 2020	13,829	Men = 3328 (24.1%) Women = 10,434 (75.5%) Other = 67 (0.5%)	Age 50+ = 7344 (53.1%)	Medium quality (++)
Fisher* et al,* 2022 [45]	South Africa	11th May–22nd May 2020	353	Men = 187 (53%) Women = 165 (46.7%) Other = 1 (0.3%)	Not reported	Acceptable (+)
Guerin* et al,* 2021 [46]	USA	10th June–25th June 2020	2565	Men = 1386 (54.0%) Women = 1179 (46.0%)	Age 50+= 1198 (46.7%)	Medium quality (++)
Hagen* et al,* 2022 [47]	Norway	1st April–2nd June 2020	19,372	Men = 4648 (24.0%) Women = 14,601 (75.4%) Other = 119 (0.6%)	Not reported	Acceptable (+)
Hammarberg* et al,* 2020 [48]	Australia	3rd April–2nd May 2020	13,762	Men = 3328 (24.2%) Women = 10,434 (25.8%)	Age 50+ = 7322 (53.2%)	Medium quality (++)
Haynes* et al,* 2021 [49]	USA	8th May–6th June 2020	276	Men = 55 (20%) Women = 221 (80%)	Age 45–64 = 49% Age 65+ = 14%	Unacceptable (0)
Hoffmann* et al,* 2023 [50]	Poland	1st June–31st Dec 2021	1306	Men = 290 (22.21%) Women = 1016 (77.79%)	Not reported	Acceptable (+)
Jewell* et al,* 2020 [51]	USA	7th April–1st June 2020	1083	Men = 189 (17.6%) Women = 884 (82.4%)	Age 45+ = 536 (50%) Age 60+ = 223 (20.7%)	Acceptable (+)
Killgore* et al,* 2021 [52]	USA	28-h period between 9th and 10th April 2020	1013	Men = 446 (43.6%) Women = 567 (56.4%)	Age 50+=160 (15.8%)	Acceptable (+)
Mani* et al,* 2023 [53]	India	25th April–10th May 2020	2640	Men = 1609 (61%) Women = 1031 (39%)	Age 51+ = 389 (14.5%)	Acceptable (+)
McDowell* et al,* 2021 [54]	USA	3rd April–7th April 2020	2301	Men = 784 (44%) Women = 1519 (66%)	Not reported	Medium quality (++)
Mojtahedi* et al, *2021 [55]	UK, Ireland, North America, India, Brazil, and others	Data collection performed on: 23rd April–21st May in UK and Ireland And 18th–25th May 2020	723	Men = 315 (43.6%) Women = 407 (56.3%) Other = 1 (0.1%)	Not reported	Acceptable (+)
Monnig* et al,* 2023 [56]	USA	18th June–19th July 2020	1079	Men = 536 (49.7%) Women = 536 (49.7%) Missing = 7 (0.6%)	Age 50+=304 (28.2%)	Acceptable (+)
Nelson* et al,* 2020 [57]	US, Canada, Europe	19th March–10th April 2020	2065	Men = 636 (30.8%) Women = 1429 (69.2%)	Not reported	Acceptable (+)
Okafor* et al,* 2021 [58]	USA	15th August–15th September 2020	446	Men = 170 (38.6%) Women = 270 (61.4%) Other = 6	Not reported	Medium quality (++)
Pieh* et al,* 2020 [59]	UK	21st April–1st May 2020	1006	Men = 462 (45.9%) Women = 544 (54.1%)	Age 55–64 = 173 (17.2%) Age 65+ = 148 (14.7%)	Acceptable (+)
Prata Ribeiro* et al,* 2021 [60]	Portugal	18th March–18th April 2020	1626	Men = 397 (24.4%) Women = 1229 (75.6%)	Not reported	Medium quality (++)
Ruengorn* et al,* 2021 [61]	Thailand	21st April–4th May 2020	2303	Men = 851 (37.0%) Women = 1384 (60.0%) Other = 68 (3.0%)	Age 51+ = 222 (9.6%)	Acceptable (+)
Settels* et al, *2023 [62]	27 European countries	June to August 2020	11,231	Men = 5161 (45.95%) Women = 6070 (54.06%)	All aged 50+	Medium quality (++)
Shahaj* et al,* 2023 [63]	27 European countries	June to August 2020	44,841	Men = 18,596 (41.5%) Women = 26,245 (58.5%)	All aged 50+	Medium quality (++)
Shalaby* et al,* 2022 [64]	Canada	24th April–2nd June 2021	186	Men = 27 (14.5%) Women = 159 (85.5%)	Not reported	Acceptable (+)
Smith* et al,* 2020 [65]	Canada	26th April – 6th June 2020	3305	Men = 1195 (36.2%) Women = 2,022 (61.2%) Missing = 88 (2.7%)	Age 55+ = 1000 (30.3%)	Medium quality (++)
Solomou* et al,* 2020 [66]	Cyprus	3rd April – 9th April 2020	1642	Men = 466 (28.4%) Women = 1176 (71.6%)	Age 50+ = 271 (16.5%)	Acceptable (+)
Umucu* et al,* 2021 [67]	USA	May–June 2020	5791	Men = 2399 (25.9%) Women = 3367 (63.1%)	Not reported	Medium quality (++)
Warren* et al,* 2021 [68]	USA	22nd June–5th July 2020	5022	Men = 2042 (40.7%) Women = 2960 (58.9%)	Age 55+ = 2195 (43.7%)	Medium quality (++)
Wright* et al,* 2021 [69]	UK	1st April–12th May 2020	41,909	Not reported	Not reported	Medium quality (++)
Zamanzadeh* et al,* 2023 [70]	China, Italy, Japan, South Korea, the UK, the US	15th April–23rd April 2020	6089	Men = 2951 (48%) Women = 3138 (52%)	Age 56–65 = 948 (16%) Age 66+ = 1035 (17%)	Acceptable (+)
Zhang* et al,* 2022 [71]	27 European countries	June to August 2020	11,197	Men = 5088 (53%) Women = 6109 (47%)	All aged 50+	Medium quality (++)
Zhao* et al,* 2021 [72]	Hong Kong	9th–23rd April 2020	1501	Men = 672 (44.8%) Women = 829 (55.2%)	Age 50+ = 748 (49.8)	Medium quality (++)
Longitudinal studies						
Batterham* et al,* 2021 [73]	Australia	Baseline data collected from 28th to 31st March 2020, and 7 follow-up online surveys up to June 2020	1296	Men = 647 (49.9%) Women = 649 (50.1%)	Age 55+ = 435 (33.6%)	Medium quality (++)
Blomqvist* et al,* 2023 [74]	Sweden	The first questionnaire between January/February 2021; follow-up between January/February 2022	1558	Men = 628 (40%) Women = 930 (60%)	Not reported	High quality (+++)
Blomqvist* et al,* 2023 [75]	Sweden	The first questionnaire between January/February 2021; follow-up between January/February 2022	1231	Men = 531 (43.1%) Women = 700 (56.9%)	Not reported	Medium quality (++)
Dragano* et al,* 2022 [76]	Germany	30th April–15th May 2020	161,787	Men = 77,773 (48.1%) Women = 84,014 (51.9%)	Age 50+ = 103,184 (63.8%)	High quality (+++)
Hwang* et al,* 2023 [77]	South Korea	3 surveys sent in: June, September and December 2020	3000	Men = 1711 (57.0%) Women = 1289 (43.0%)	Age 50+ = 17.1%	Acceptable (+)
Matsubayashi* et al,* 2022 [78]	Japan	June 2020–February 2021	9000	Men = 4464 (49.6%) Women = 4536 (50.4%)	40–64 years: Men—1629 (36.49%), Women—1620 (35.71%), Total—3249 (36.1%)	Acceptable (+)
Savolainen* et al,* 2021 [79]	Finland	Baseline information gathered in September–October 2019. Follow-up data in September–October 2020	1044	Men = 572 (54.79%) Women = 472 (45.21%)	Age 50–66 = 405 (38.79%)	High quality (+++)
Yao* et al,* 2021 [80]	USA	Baseline survey on 23rd April 2020, followed by follow-up surveys for up to 17 weeks	1,576,770	Men = 762,684 (48.37%) Women = 814,086 (51.63%)	Age 50+=735,411 (46.6%)	Acceptable (+)
Zhou* et al,* 2020 [81]	USA	Wave 1: 20th April 2020 Wave 2: 4th–8th May Wave 3: 18th–22nd May	1021	Men = 483 (47.31%) Women = 534 (52.30%) Non-binary = 4 (0.39%)	Age 50+ = 414 (40.5%)	Medium quality (++)

*GAD-7* Generalised Anxiety Disorder Assessment – 7 items, *GAD-2* Generalised Anxiety Disorder Assessment – 2 items, *DASS-21* Depression, Anxiety, Stress Scale – 21, *STAI* State-Trait Anxiety Inventory, *BAI* Beck Anxiety Inventory, *AOR* Adjusted Odds Ratio, *RR* Relative Risk, *WFH* Working From Home

The main reasons for scoring poorly on quality assessment were as follows: recruitment of participants mainly performed with snowball techniques which do not ensure representativeness of the sample; the use of a cross-sectional design which prevents inference about causality; and failure to adjust for important confounders in the analyses such as a measure of socio-economic status and a measure of health. Only a minority of studies used data from established cohorts recruited pre-pandemic [62, 63, 71, 74–76, 79]. Of the cross-sectional studies, the majority (*n* = 21) were rated “acceptable”, 17 “medium quality” and 1 “unacceptable”. Longitudinal studies tended to be of higher quality and 3 were rated “acceptable”, 3 “medium quality” while 3 “high quality”. Details of the quality assessment for each study are provided as supplementary material (Additional File 4).

### Main findings of descriptive studies

Table 2 shows the main findings from those descriptive studies which were excluded from the “vote-counting” process. These were all cross-sectional in design. The quality was rated “acceptable” in eight and “unacceptable” in one [49]. Five of the eight descriptive studies described anxiety among those who experienced job loss. The studies by Abdalla [82] and Killgore [52] reported that the prevalence of anxiety was significantly higher among participants who lost their job since the start of the pandemic as compared with those who did not. In a sensitivity analysis conducted by Killgore et al. based on 16% of their sample aged 50+, the authors found comparable results to those reported in the whole sample. Amer et al. [37] surveyed 859 adults living in Saudi Arabia, and reported that anxiety score was higher for participants with work suspension, as opposed to those working as before, and similar findings were reported by Pieh in the UK [59]. On the contrary, a study conducted in a deprived neighbourhood of Johannesburg (South Africa), found no differences in the rate of anxiety between those who lost their jobs and those who did not [45].

**Table 2:** Main findings of descriptive studies

Author	Exposure definition	*N* (%) with exposure	Outcome definition	*N* (%) with outcome/Mean (SD)	Main findings	Main findings age 50+
Abdalla *et al* [34]	Job loss	Not mentioned	GAD-7 with a cut-off 15.	Prevalence 10.9% (95% CI 9.1% to 13.2%)	Prevalence of probable anxiety significantly higher among participants who lost their jobs because of COVID-19 (24%) compared with those who did not (9%) (*p*<0.05)	
Amer* et al * [37]	Working remotely, decreased working hours, work suspension or working as before the pandemic.	Working as before= 381 (44.5%) Working remotely=199 (23.2%) Decreased working hours=191 (22.3%) Work suspension = 86 (10.0%)	GAD-7 analysed as continuous.	Breakdown by anxiety categories No anxiety: 260(30.2); Mild: 355 (41.2); Moderate: 162 (18.8); Severe: 84 (9.8) Mean GAD-7=4.01; SD=4.9; median=2, range (0–21)	Anxiety score was higher for participants working remotely or with work suspension, as opposed to those working as before. Median (mean and SD) Working as before: 2(3.9 SD=4.9) Working remotely: 3(4.5 SD=4.9) Work suspension: 3(4.8 SD=5.5)	
Elezi* et al * [42]	In work but employment interrupted by the pandemic, work from home after the pandemic, continue to go to work like before.	In work but employment interrupted by the pandemic: 262 (15.6%) Work from home after the pandemic: 309 (18.4%) Continue to go to work like before pandemic: 338 (20.1%)	GAD-7 analysed as continuous.	Not specified	Mean (SD) GAD-7. In work but employment interrupted by the pandemic: mean 5.74 (5.098) Work from home after the pandemic: mean 5.06 (4.364) Continue to go to work like before pandemic: mean 4.74 (4.423). Those who kept working as before had the lowest anxiety score	
Fiorenzato* et al* [43]	Working condition under lockdown as follows: Working outside home, working from home, underemployed.	Working outside home = 297 (24.4) Working from home = 535 (44.0) Underemployed = 383 (31.5)	Hospital Anxiety and Depression Scale - Anxiety (HADS-A) used to assess the presence of anxiety. Cut-off score of 8 used to identify clinically significant disturbances.	Total N (%) HADS-A>8 = 434 (35.72%) Mean (SD) HADS-A = 6.51 (4.03)	Mean difference in anxiety between underemployed and WFH = 0.73 (95%CI 0.22 to 1.24, *p*<0.005). The same comparison WFH vs outside was not significant and not reported.	
Fisher* et al * [45]	Job loss. Those not in work before lockdown were excluded.	Prevalence of job loss = 70.1% (95%CI = 64.1, 76.1)	Participants were asked how often in the past week they felt anxious or stressed. Answers were: never/some of the time/most of the time/all of the time and dichotomised as yes/no.	Prevalence anxiety overall = 82.0 (78.0, 86.0) Women = 83.5 (77.8, 89.2) Men = 80.6 (74.9, 86.3) Age ≥50 years = 66.0 (53.2, 78.8)	Similar proportion reported anxiety among those who lost a job (83.1 (77.2, 89.0)) vs those who did not (83.1 (74.0, 92.2)). No significant differences found between the groups (*p*=0.99)	
Haynes* et al * [49]	Not employed outside home prior to COVID-19, switched to home working, continued working outside the home. Those who lost their job were excluded.	Not employed outside home prior to COVID-19 = 59(21.4%); Switched to home working = 111(40.2%) Continued working outside the home= 89 (32.2%)	Unclear how anxiety was assessed	Prevalence of anxiety = 52%	Those continuing work outside home reported high levels of anxiety (52%), as well as those who switched to remote working (54%). Those who did not work outside home prior to lockdown reported slightly lower anxiety (49%). *P*-value not reported.	
Killgore* et al* [52]	Job loss since COVID-19	Total job loss = 176 (17.4%), Men = 66 (14.8%), Women = 110 (19.4%)	Anxiety defined as GAD-7>8, Zung Self-rated Anxiety Scale (SRAS) > 36; the state and trait portions of the Spielberg State-Trait Anxiety Inventory (STAI) > 46.	Total with anxiety based on GAD-7 *N* (%) = 321 (32.7); Zung SRAS=462 (45.8); STAI-state=302 (29.8); STAI-trait=402 (39.7). Mean (SD) of GAD-7=6.0 (5.8); Zung SRAS = 36.2 (9.4); STAI-state = 40.3 (11.0); STAI-trait = 41.7 (13.1)	Mean anxiety significantly higher in the group with COVID-19 job loss vs the group without job loss. Also, the proportion of participant who reach a clinically meaningful anxiety is significantly higher among those with job loss.	The proportion of participants with clinically meaningful anxiety is higher among those with job loss vs no job loss.
Pieh* et al * [59]	Not working now and did not work before, not working now but was working before lockdown, home office, working in the usual place, reduced working hours, retired.	Not reported.	GAD-7 with a cut-off 10.	Total *N* (%) = 392 (39.0), Men = 144 (31.2), Women = 248 (45.6), Aged 55–64 = 52 (30.1), Aged 65+ = 18 (12.2)	Prevalence of anxiety is 46% among those who lost their job vs 40.6% among those with unchanged work vs 36.9% among those working remotely. Chi-squared test across all categories of work status is significant with *p*<0.001	
Solomou* et al * [66]	I am working from home, I sometimes work from home and sometimes at my workplace, I’m still working at my workplace, I’m out of work and will be paid 60% of my salary, other. Those not in employment were excluded.	I am working from home = 480 (29.2%) I am still working at my workplace = 178 (10.8%) I’m out of work and will be paid 60% of my salary = 49 (3.0%)	GAD-7 and analysed as continuous	Normal = 589 (35.9%) Mild = 673 (41%) Moderate = 230 (14%) Severe = 150 (9.1%) Mean GAD-7 overall = 6.79 (SD=4.74) Mean GAD-7 Men = 5.21 (SD=4.18) Mean GAD-7 Women = 7.42 (SD=4.80)	No significant difference in the GAD-7 score across categories of work status according to the Kruskal-Wallis test. Working from home = mean GAD-7 6.11 (SD=4.34) Working at the workplace = mean GAD-7 6.57 (SD=5.11) Out of work and paid 60% = mean GAD-7 6.77 (SD=4.64)	

*GAD-7* Generalised Anxiety Disorder Assessment – 7 items, *WFH* work from home

Six of these studies reported on the prevalence of anxiety among those who were working from home since lockdown. In most of these studies, participants working from home since lockdown were not dissimilar regarding their prevalence of anxiety, compared with those whose employment had remained unchanged. This was reported in studies from Italy [43], the UK [59], the USA [49] and Cyprus [66]. On the contrary, Amer et al. [37] and Elezi et al. [42] both reported that participants working from home in lockdown had a higher mean level of anxiety compared with those who remained working as before.

### Main findings of analytical studies

#### Job loss and anxiety

Similarly, Table 3 describes the main findings from the 39 analytical studies. All nine longitudinal studies explored the prospective association between job loss and anxiety. A study performed in Australia found no association between loss of employment and clinically significant anxiety [73]. The study by Matsubayashi et al. [78] collected data on 9000 residents in Japan and found that experiencing an adverse job change of any kind (including but not limited to job loss) was associated with increased odds of anxiety. The study by Savolainen and colleagues [79] collected data in 2019 and 2020 on a representative sample of 1044 Finnish workers and found no increase in anxiety for those who became unemployed since lockdown. Yao and colleagues [80] showed that participants involuntarily not working (vs those still in work) were 20% more likely to report anxiety and that any reason for involuntarily not working (being laid off, employer’s business closure due to COVID-19, employer went out of business) was associated with significantly increased odds of anxiety as compared with people voluntarily not working (i.e. retirees). Zhou et al. [81] recruited 1021 participant residents in the USA who completed two surveys 1 month apart. Participants either laid-off, furloughed or otherwise unemployed due to COVID-19 did not display different levels of anxiety compared with participants already unemployed pre-pandemic. Blomqvist et al. published two papers which feature in this review. They analysed participants of an existing cohort of working age Swedes to investigate the prospective association between job loss (as opposed to a stable work situation) and anxiety [74] and between having been dismissed or received notice and anxiety [75]. In both studies, they found no significant associations of anxiety with change of job status after full adjustment. In a large population-based German cohort, although only less than 1% of the sample reported unemployment due to COVID-19, the authors showed that having such experience led to an increase in anxiety (as opposed to no change in employment position) [76]. Finally, a study in South Korea, where 15% of the cohort experienced job loss, showed a negative association between job loss and anxiety [77]. The remaining studies to explore such associations were of cross-sectional design. These studies can be divided into those showing significant negative effects of job loss on anxiety [36, 40, 44, 46, 47, 57, 64, 70, 83] and those finding no significant effects between the two [41, 56, 61, 69].

**Table 3:** Main findings of the analytical studies, by type of study design

Author	Exposure definition	N (%) with exposure	Outcome definition	N (%) with outcome or Mean (SD)	Confounding factors	Estimates (95%CI)	Estimates (95%CI) age 50+
CROSS-SECTIONAL STUDIES							
Abrams* et al.* [35]	Job transition since the pandemic: lost employment, furloughed, reduced hours or income, and work-from-home.	Work unchanged: 509 (8.12%) Not working, unchanged: 4303 (68.7%) Lost job: 116 (1.85%) Furloughed: 359 (5.73%) Reduced hours or income: 447 (7.13%) Work from home: 531 (8.47%)	Anxiety symptoms assessed with the 5-item Beck Anxiety Inventory (BAI)	Mean (SD) anxiety symptoms: 1.67 (SD = 0.60)	Gender, age, race/ethnicity, educational attainment, occupation, medical condition, use of mobility aid, smoking, living alone, household membership, relationship status, US Census division, prior diagnosis of anxiety		All participants aged 55+ Reference: work unchanged Job loss β(95%CI) 0.23(−0.02,0.49) Furloughed β(95%CI) 0.07 (−0.10,0.25) Reduced hours or income (95%CI) 0.06 (−0.09,0.22) Work from home β(95%CI) 0.14 (−0.00,0.28)
Alsaif* et al* [36]	Job loss	Total job loss = 51 (6.8%)	Arabic version of the Depression Anxiety Stress Scale (DASS-21).	Total with anxiety of any severity = 34.8%	Sex, age, nationality, educational level, marital status, chronic health issues, diagnosed with COVID-19	Job loss vs not AOR (95%CI) 2.02 (1.10, 3.74) *p*<0.05	
Burhamah* et al * [38]	Working or studying from home, work suspended, attending work as usual, retired prior to lockdown, unemployed prior to lockdown.	Stopped working/studying = 1620 (39.2%)	GAD-7 with a cut-off score of 8.	GAD-7≥8 = 1086 (26.3%)	Gender, age, working health sector, risk of getting virus at work, past psychiatric history, home quarantine, rating of the government protocol, time on social media, time following COVID-19 news	Stopped working or studying vs Working or studying from home OR (95%CI) *p*-value 1.39 (1.04–1.86) 0.026	
Burstyn* et al * [39]	Job loss, working hours remained the same, increased or decreased since the pandemic.	Lost job=67(7.4%), Men=14, Women=53	Hospital Anxiety and Depression Scale (HADS) to measure anxiety with score ranging from 0 to 21. HAD-A analysed as continuous. HAD-A≥11 indicates anxiety	HAD-A≥11: Men = 15(19%); Women = 78 (40%)	Age, education, income, children living at home, phase of stay-at-home order, general health	Lost job vs same working hours MEN RR (95%CI) 1.56 (1.12, 2.19) Lost job vs same working hours WOMEN RR (95%CI) 0.94 (0.78, 1.13)	
Dawel* et al * [40]	Job loss because of COVID-19; Working from home due to COVID-19	Total job loss = 117 (9.0%) Men job loss = 50 (7.8%) Women job loss = 67 (10.3%) Total WFH = 173 (13.4%) Total WFH Men= 78 (12.1%) Total WFH Women = 95 (14.6%)	GAD-7 used as continuous score	Mean GAD-7 Overall 4.4 (SD=5.2) Men 3.7 (SD=4.9) Women 5.1 (SD=5.4) GAD-7≥ 10 =212 (16.4%)	Age, gender, education, has partner, lives alone, child at home, any chronic disease, any neurological disorder, any current MH disorder, bushfire exposure to smoke, fire, other adverse life event, COVID-19 exposure, financial distress, work and social adjustment scale (all factors significant in univariate analyses)	Job loss (vs not) (multivariate) β *p*-value 0.51 0.665 WFH (vs not) (univariate) β (SE) *p*-value 0.18 (0.42) 0.665	Job loss (vs not) (multivariate) β (95%CI) *p*-value 0.608 (−1.053, 2.269) 0.473 WFH (vs not) (multivariate) β (95%CI) *p*-value 0.193 (−1.099, 1.485) 0.769
De Miquel* et al * [41]	Unemployed or temporarily laid-off (furlough) due to the coronavirus pandemic.	Unemployed or temporarily laid-off: *n*=64, 26.93% (25.10–28.83%)	GAD-7 with a cut-off score of 10.	GAD-7≥10= 272 (11.4%)	Gender, age, education level, and marital status	Unemployed or temporarily laid-off (vs not) AOR (95%CI) 0.93 (0.69–1.26)	Unemployed or temporarily laid- off (vs not) AOR (95%CI) 1.47 (0.82–2.63)
Fisher* et al * [44]	Job loss because of restrictions	Job loss: 1251(9.0%)	GAD-7 with a cut-off score of 10.	GAD-7≥10 = 3661, 21.0% (95%CI 19.6–22.4%)	Any COVID experience, worried about COVID, great impact of restrictions, and state, remoteness and socio-economic quintile of residence, sex, age, living situation, place of birth, and employment status.	Job loss (vs not) AOR (95% CI) 1.22 (1.06–1.41)	
Guerin* et al * [46]	Job loss, temporarily laid off or furloughed, working hours reduced, No change in position	Job loss = 108 (4.2%) Temporarily laid off or furloughed = 317 (12.4%) No change = 1502 (58.6%)	GAD-2 used as continuous variable.	Not reported	Age, sex, race, education, marital status, social support, household income, interaction between job loss and income.	Job loss (vs not) β (SE) *p*-value 1.08 (0.43). 0.013 Hours reduced (vs not) β (SE) *p*-value 0.88 (0.30) 0.003	Job loss (vs not) β (SE) *p*-value 0.49 (0.79). 0.53
Hagen* et al * [47]	Job loss	Job loss = 411 (2.1%)	GAD-7 used as continuous variable.	Minimal 44.2%; Mild 32.1%; Moderate 15.0%; Severe 8.7% Mean GAD-7 = 6.28 (SD=5.07)	Infection self/family, sex, student, mental health problems, increased use of alcohol, or tobacco, less exercise, economic impact, lower education.	Job loss (vs not) Standardised β *p*-value 0.03 <0.001	
Hammarberg* et al* [48]	Job loss because of COVID-19 restrictions	Job loss Men=277 (8.3%) Women=964 (9.2%)	GAD-7 with a cut-off score of 10.	GAD-7≥10: Men = 472(14.2%) Women = 275(21.8%)	Deprivation quintile, living situation, caring for children, caring for relatives, worried about contracting COVID-19, high adverse impact of restrictions		Job loss (vs not) Women age 50+ OR (95%CI) 1.56 (1.20–2.02) Job loss (vs not) Men age 50+ OR (95%CI) 1.38 (0.83–2.29)
Hoffmann* et al* [50]	Place of work during the COVID-19 pandemic: hybrid, in the workplace, or remotely at home.	Hybrid = 211 (28.63) In the workplace = 377 (51.15) Remotely at home = 149 (20.22)	DASS-21	Mean (SD) DASS-21=7.25 (6.53)	Only univariate associations reported	Remotely at home vs unemployed β (95%CI); *p*-value −0.30 (−1.64, 1.03) 0.655	
Jewell* et al * [51]	Working remote before and after COVID-19 Unemployed prior to COVID-19 Work outside home No longer working due to COVID-19 Working remotely due to COVID-19	Working remotely before and after COVID-19: 104 (9.9%) No longer working due to COVID-19: 107 (10.2%) Working remotely due to COVID-19: 552 (11.4%)	GAD-7 with a cut-off score of 10.	GAD-7≥10 = 342 (34.0%)	Age, race, ethnicity, insurance, gender, household size, 5 measures of concern (financial, food access, economy, illness, death)	No longer working due to COVID-19 (vs working remotely before and after COVID-19) OR *p*-value 1.32; *p* 0.45 WFH (vs working remotely before and after COVID-19) OR *p*-value 0.70; *p* 0.22	
Mani* et al * [53]	Temporary unemployed, forced to work from home	Temporary unemployed = 499 (22%) Forced to work from home = 975 (37%)	GAD-7 with a cut-off score of 10.	Mean GAD-7 = 2.71 (SD=4.33). Moderate anxiety = 114 (19%); severe anxiety = 87 (14%). Men = 324 (20.1%); Women=284 (27.6%); Age 50+ = 66 (17%)	Sex, age, lack of salaried jobs, work stress, being a healthcare worker, media reports.	Temporary unemployed (vs still employed) AOR *p*-value 2.02; *p*<0.001 Prevalence of anxiety lower among those WFH (22.6%) vs. away from home (28.4%)	
McDowell* et al * [54]	No change in work, working from home, when I was not before, and lost employment in relation to pandemic.	No changes: 34% Started working from home: 54% Lost job: 12%	21-item Beck Anxiety Inventory (BAI) and analysed as continuous.	Mean ± SD BAI: 7.96 ± 8.38	Age, sex, race, BMI, smoking, screen time, physical activity, marital status, chronic conditions, public health restrictions.	Hedges’ g (95%CI) Job loss (vs no change in work) Hedges’ g (95%CI) *p*-value-0.212 (−0.363 to −0.061) 0.008	
Mojtahedi* et al * [55]	Unemployed before the pandemic, I lost my job/business during the pandemic, furloughed, I still have my job/business and travel to work, I still have my job/business and working from home.	Total job loss = 64(9.1%); sample A: 14(3.9%) sample B: 50 (14.4%) Furloughed = 106(15.1%); sample A: 75 (21%) sample B: 31 (8.9%) Previously unemployed = 109(15.5%); sample A: 63(17.6%) sample B: 46(13.3%) Working (travelling) = 146(20.7%); sample A: 80(22.4%) sample B: 66(19%) Still in work and working (home) = 279(36.9%); sample A: 125(35%) sample B: 154(44%)	DASS21 and STAI used as continuous.	Participants with moderate/severe anxiety: 117(31%) in sample A; 221 (63.7%) in sample B	Challenge, commitment, control, and confidence	Job loss (vs WFH) DASS21 β with *p*-value 0.13 *p*<0.001 Furlough (vs WFH) DASS21 β −0.03 Working travelling (vs WFH) DASS21 β with *p*-value 0.07 *p*<0.05 Job loss (vs WFH) STAI β with *p*-value 0.009 *p*<0.001 Furlough (vs WFH) STAI β −0.001 Working travelling (vs WFH) STAI β 0.02	
Monnig* et al * [56]	“Have you, or has anyone in your household experienced a loss of employment since March 13, 2020”?	Personal/household loss of employment = 447 (41.4%)	GAD-7 with a cut-off score of 10.	Mean (SD) of GAD-7 = 7.0 (5.6). GAD-7≥10 = 34.8%	Age, household income, living alone, education, race, ethnicity, gender, know someone hospitalised, worried about covid, essential worker status, children in the household, covid testing history, loneliness, barriers to environmental rewards, food insecurity.	Loss of employment (vs not) AOR (95%CI) 1.36 (0.93, 2.05)	Loss of employment (vs not) OR (95%CI) 3.007 (1.533, 6.917)
Nelson* et al * [57]	Not clearly defined. “Measure of COVID-associated financial strain included questions associated with lost or change in job, income, and financial comfort”	Job loss = 280 (13.56%)	GAD-2 with a cut-off 3.	Mean GAD-2 score = 3.31 (SD=1.97)	Gender, age, and date completion questionnaire	Job loss (vs not) β (95%CI) *p*-value 0.227 (−0.023,0.476) *p*=0.076	
Okafor* et al * [58]	Lost job or wages because of COVID-19	Lost job or wages = 113 (26.0)	“Since the outbreak I feel negative and/or anxious about the future”. Responses ranging from “strongly agree” to “strongly disagree”. Binary variable: agree vs disagree	284 (65.0%)	Age, sex, education, marital status, health insurance, overall health, family member has covid, financial difficulties, smoking, smoked more (vs not), drank more (vs not).	Lost job or wages (vs employed during the outbreak) OR (95% CI) *p*-value 3.92 (2.07,7.44)	
Prata Ribeiro* et al * [60]	working from home, working at workplace, not working in lockdown	Working from home = 922 (56.7%) Working at workplace = 262 (16.1%)	Portuguese version of the Beck Anxiety Inventory (BAI) and analysed as continuous.	N (%) with at least mild symptoms = 864 (53.1%) Mean BAI = 10.2 (SD=8.2).	Age, gender, occupation, days in isolation, contact with COVID-19, under psychiatric care, receiving psychiatric medication	WFH (vs in the workplace) β (95%CI) *p*-value −1.66 (−2.86, −0.46) 0.007	
Ruengorn* et al* [61]	Job loss since the COVID pandemic	Job loss Total = 262 (11.4%) Men = 77 (9.0%) Women = 178 (12.9%) Age ≥51 years = 12 (5.4%)	Thai version of the Generalised Anxiety Disorder Scale—7‐items (GAD‐7). A cut-off of 5 was used to identify those with anxiety symptoms	GAD-7≥5 = 944 (41.0%)	Age, sex, marital status, education, religion, region, living status, reimbursement scheme, history mental illness, history NCD, debt, exposure during outbreak, confirmed cases in community, quarantine status, resilient coping	Job loss (vs not) OR (95%CI) *p*-value 1.39 (0.89–2.18) 0.146	
Settels* et al * [62]	“Due to the Corona crisis have you become unemployed, laid off or had to close your business?” Those not in employment immediately before the pandemic were excluded.	Lost employment = 2079 (18.51%)	“In the last month, have you felt nervous, anxious or on edge?” (yes/no)	Anxiety symptoms = 2999 (26.7%)	Gender, age, self-rated health before the COVID-19 era, education, country, loneliness, household making ends meet, face-to-face contact with non-relatives.		Lost employment (vs not) AOR (95%CI) *p*-value 1.567 (1.169, 2.099) <0.001
Shahaj *et al * [63]	Job loss due to COVID-19	Job loss = 1726 (3.8%)	Participants were asked if in the preceding 4 weeks they felt nervous. If they answered yes, they were asked whether symptoms had worsened. Outcome is a binary variable with a value of 1 if “more nervous”, a value of 0 if “remained the same or improved”	More nervous = 9725 (70.7%) Analyses on this outcome are based on a smaller sample of *N*=13,755	Sex, age, education, having a partner, living alone, multimorbidity, worsening health, someone hospitalised with COVID-19, someone died from COVID-19, frequency of social contact, country-level variables (COVID-19 deaths, stringency index, GDP, GINI)		Job loss (vs not) AOR (95%CI) *p*-value 2.06 (1.49, 2.85) <0.001
Shalaby* et al * [64]	Job loss due to COVID-19	Job loss=21 (12.1%)	GAD-7 with a cut-off score of 10.	GAD-7≥10 = 71 (42.5%)	Not employed, depression diagnosis, mental health counselling, would like mental health counselling, medication for mental health concerns, no support from family, no support from Government, no support from employer	Job loss (vs not) AOR (95%CI) *p*-value 4.401 (1.007–19.241) *p* 0.049	
Smith* et al * [65]	Working remotely, working at workplace, no longer employed	Working remotely: 1376 (41.6%) Site-based workers: 1693 (51.2%) No longer employed: 236 (7.1%)	GAD-2 with a cut-off score of 3.	GAD-2≥3=1399 (42.3%)	Age group, sex, visible minority status, disability, population density, province of residence, supervisory status, job tenure, co-workers with COVID-19, experiencing symptoms of COVID-19, being exposed to someone with COVID-19, workplace size, date of survey	Adjusted proportion (95% CI) Working remotely =35.3 (27.1–43.5) Site-based workers =43.5 (35.4–51.6) No longer employed=43.8 (34.0–53.7) Site based with 100% PPE =33.9 (25.0–42.7) Site-based workers with 100% ICP =29.8 (20.5–39.0)	Age group 55+, adjusted proportion (95% CI) Working remotely = 26.2(20.2–32.2) No longer employed = 28.1 (17.8–38.5) Site-based = 34.5 (28.5–40.5) Site-based with 100% ICP = 20.6 (12.1–29.1) Site-based with 100% PPE = 23.6 (15.9–31.3)
Umucu* et al * [67]	Job loss in the past month	Job loss=598 (9.0%)	Probable anxiety (yes/no) measured with Patient Health Questionnaire (PHQ-4)	Participants with probable anxiety 2116 (30.0%)	Adjustment for age, sex, race, education, government response to COVID-19, viewing COVID-19 as a threat to Americans, probable depression	Job loss (vs not) OR (95%CI) *p*-value 1.48 (1.21–1.81) *p*<0.01	
Warren* et al * [68]	Working from normal location, working from home, not working right now due to COVID19, unemployed right now due to COVID-19, not working for other reasons” (e.g. as a student) unrelated to COVID-19.	Working from normal location = 2539 (50.5%) Working from home = 1256 (25.0%) Unemployed due to COVID-19 = 227 (4.5%)	GAD-7 used as continuous and as categorical (with a cut-off score of 10).	GAD-7≥10 = 716 (14.3%)	Age, sex, race, marital status, education, current psychological diagnosis, log COVID-19 case and death count per 100k county population.	Work from home (vs normal location) β (95%CI) *p*-value 0.36 (−0.03,0.75) 0.096 Unemployed due to COVID-19 (vs normal location) β (95%CI) *p*-value 2.49 (1.53,3.44) <0.001 Work from home (vs normal location) OR (95%CI) *p*-value 1.16 (0.93,1.45) 0.262 Unemployed due to COVID-19 (vs normal location) OR (95%CI) *p*-value 2.78 (1.86,4.16) <0.001	
Wright* et al * [69]	Job loss or been unable to do paid work	Total mean lost work = 0.10 (SD=0.30)	GAD-7 used as continuous.	Total average GAD-7 score = 4.67 (SD=5.24)	Worries about employment, day of the week, days since lockdown began + other time constant variables (these are supposedly socio-economic, personality and other variables that do not vary over time). Unclear which ones were included	Standardised β (95%CI) Employment adversities experiences (vs not)/predicted change over time in anxiety = 0.034 (−0.012, 0.079)	
Zamanzadeh* et al * [70]	“Have you lost your job or has your activity (as self-employed) been stopped as a consequence of the COVID-19 pandemic?”	Job loss or activity stopped = 1835 (30.0%)	“Have you experienced any anxiety due to the COVID-19 pandemic?	Total N (%) = 2780 (46%)	Mortgage, gender, age, income, living area, religious services.	Marginal effect job loss Probability (SD) *p*-value 0.055 (0.015); *p*<0.001	
Zhang *et al * [71]	Since lockdown, worked at home only/ worked at the usual place/worked from home and at the usual place/ none of these. Participants retired, unemployed or who were laid off were excluded.	Proportion and 95%CI Worked at the usual place = 5809 (52.1 (49.8, 54.5)) Worked from home only = 1848 (14.3 (12.8, 15.9))	“In the last month, have you felt nervous, anxious, or on edge?”	Total, N (proportion and 95%CI) More nervous than before = 2306 (21.1 (19.1, 23.2))	Age, gender, education level, live alone, contact less often or never, household income, change working hours, difficulty with daily activities, chronic disease, vaccination, close to suspected or confirmed COVID-19 cases		WFH (vs usual place) AOR (95%CI); *p*-value 1.40 (0.87, 2.27); 0.1656
Zhao* et al * [72]	Reduction in income since the outbreak (no change, small reduction, reduction by half, larger reduction, or unemployed). Students, retirees, and homemakers excluded.	Became unemployed = 70 (6.7%)	GAD-2 with a cut-off score of 3.	GAD-2≥3 = 218 (14.5%)	Sex, age, and education, PPE, social distancing measures	Unemployed (vs no change in income) OR (95%CI) *p*-value 5.38 (2.64–10.96) *p*<0.001	
LONGITUDINAL STUDIES							
Batterham* et al * [73]	Lost job; work from home	Lost job = 117(9.0) Work from home = 173(13.3)	GAD-7 with a cut-off score of 10 and used as continuous.	GAD-7≥10 at each data point 212(16.4%) 164 (16.9%) 163 (17.2%) 137 (15.1%) 112 (12.9%) 104 (12.8%) 102 (13.5%)	Unclear	Lost job (vs not)/baseline anxiety β (SE) *p*-value 0.025 (0.423) 0.92 Lost job (vs not)/linear change anxiety β (SE) *p*-value 0.243 (0.246) 0.32 Lost job (vs not)/quadratic change anxiety β (SE) *p*-value −0.029 (0.039) 0.45 WFH (vs not)/baseline anxiety β (SE) *p*-value 0.293 (0.631) 0.42 WFH (vs not)/linear change anxiety β (SE) *p*-value −0.371 (0.188) 0.049 WFH (vs not) /quadratic change anxiety β (SE) *p*-value 0.056 (0.029) 0.05	
Blomqvist* et al* [74]	Stable work situation, furloughed, job loss.	Stable work situation: 1171(75%); furloughed: 140(9%); job loss: 98(6%)	GAD-7 with a cut-off score of 10.	GAD-7≥10 = 191 (14%) Mean score = 75, SD=5	Sex, age, education, country of birth, socio-economic classification, civil status, prior mental health problems	Job loss (vs stable work) AOR (95%CI) 1.18 (0.36, 3.83) Furloughed (vs stable work) AOR (95%CI) 0.66 (0.23, 1.91)	Job loss (vs stable work) AOR (95%CI) 0.57 (0.13, 2.55) Furloughed (vs stable work) AOR (95%CI) 0.88 (0.20, 3.92)
Blomqvist* et al * [75]	Dismissed or received notice, furloughed, became unemployed since the outbreak	Dismissed or received notice=45 (3.7%); furloughed=151 (12.6%)	GAD-7 with a cut-off score of 10.	GAD-7≥10 = 69 (5.6%)	Job security, sex, age, mental health at baseline	Dismissal or notice (vs not) AOR (95%CI) 0.73 (0.13, 4.00) Furloughed (vs not) AOR (95%CI) 0.68 (0.17, 2.77)	Dismissal or notice (vs not) AOR (95%CI) 3.84 (0.56, 26.35) Furloughed (vs not) AOR (95%CI) 0.66 (0.10, 4.22)
Dragano* et al * [76]	No pandemic-related changes, unemployed due to corona, working from home since the pandemic at least some days	No pandemic-related change = 68,765 (42.5%) Unemployed due to corona = 828 (0.5%) Working from home since the pandemic at least some days =44,174 (27.3%)	GAD-7 with a cut-off score of 10.	GAD-7≥10= 10161 (6.3%) Mean score (SD) = 3.40 (3.54)	Age, gender, type of household, high-risk contact with infected person, own COVID-19 infection, self-reported health	Unemployed due to corona (vs no change) β (95%CI) *p*-value 0.66 (0.44,0.88) <0.001 WFH (vs not) β (95%CI) *p*-value 0.28 (0.25, 0.32) <0.001 Unemployed due to corona (vs no change) RR (95%CI) *p*-value 1.30 (1.11, 1.51) <0.001 WFH (vs not) RR (95%CI) *p*-value 1.05 (1.00, 1.09) <0.05	Unemployed due to corona (vs no change) β (95%CI) *p*-value 0.59 (0.23,0.94) <0.001 WFH (vs not) β (95%CI) *p*-value 0.25 (0.19, 0.30) 0.001 Unemployed due to corona (vs no change) OR (95%CI) 1.58 (1.02, 2.44) WFH (vs not) OR (95%CI) 1.03 (0.94, 1.14)
Hwang* et al * [77]	Job loss	Total = 15.1%; Women = 17.9%; Men = 12.9%	Participants were asked: “how much anxiety do you feel due to the COVID-19 pandemic?”. Analysed as continuous.	Mean (SD) anxiety. Total= 2.509 (0.740) Men=2.451 (0.735) Women=2.586 (0.741)	Sex, age, education, job type, firm size, occupational characteristics, time of survey.	Job loss (vs not) β (SE); *p*-value −0.168 (0.045) *p*<0.01	
Matsubayashi* et al * [78]	Any of job loss, layoff, or reduction in working hours (=Adverse change)	Any adverse change Total = 1116 (12.4%) Men = 547 (12.25%) Women=569 (12.54%)	GAD-7 with a cut-off score of 10.	Not reported	Sex (in the analysis including the whole sample), age, survey rounds, log number of monthly total COVID-19 infections and deaths in the prefectures	Adverse change (vs not) overall AOR (95%CI) 1.838 (1.502-2.174) Men AOR (95%CI) 1.828 (1.354–2.301) Women AOR (95%CI) 1.857 (1.378–2.36)	Adverse change (vs not) overall AOR (95%CI) 1.886 (1.246–2.527) Men AOR (95%CI) 2.421 (1.331–3.512) Women AOR (95%CI) 1.406 (0.660–2.152)
Savolainen* et al * [79]	Change in employment was measured between 2019 and 2020. Became unemployed since the beginning of the pandemic. Became remote worker since the beginning of the pandemic.	Became unemployed = 3.35% Became remote worker = 12.25% Total remote worker in 2020 = 391 (37.45%)	COVID-19 anxiety assessed with the Spielberger State–Trait Anxiety Inventory STAI-6.	Participants reporting at least some anxiety = 531 (50.86%)	Model 0 = age and gender Full Model = loneliness, distress, technostress, work exhaustion, openness, consciousness, extroversion, agreeableness, neuroticism, social media information bubble, social support from work, remote work, lives alone, sex, age, income, educational level, occupational area	Cross sectional Remote worker (vs not) (full model) β (95%CI) *p*-value 0.01 (0.46) 0.98 Became unemployed (vs not) (model 0) β (95%CI) *p*-value 1.05 (1.14) 0.360 Became remote worker (vs not) (model 0) β (95%CI) *p*-value 0.41 (0.65) *p*-value 0.535	Became unemployed (vs not) (model 0) β (95%CI) *p*-value 3.17 (1.85) 0.09 Became remote worker (vs not) (model 0) β (95%CI) *p*-value 1.04 (1.05) 0.325
Yao* et al * [80]	Employed, voluntarily not working (i.e. retired), Involuntarily not working which included laid off, Employer’s business closed temporarily due to COVID-19, Employer went out of business.	Working = 930,472 (59.01%) Voluntarily not working = 307,179 (19.48%) Involuntarily not working = 339,119 (21.51%)	GAD-2 with a cut-off score of 3.	GAD-2≥3 = 503,147(31.91%)	Economic status, health, access to medical care, interview weeks, age, sex, race, education, marital status, number of children	Involuntarily not working (vs in work) OR *p*-value 1.203 <0.001 Laid off (vs voluntarily not working) OR *p*-value 1.538 <0.001 Employer’s business closed temporarily due to COVID-19 (vs voluntarily not working) OR *p*-value 1.302 <0.001 Employer went out of business (vs voluntarily not working) OR *p*-value 1.703 <0.001	Involuntarily not working (vs in work) OR *p*-value 1.169 <0.001 Laid off (vs voluntarily not working) OR *p*-value 1.504 <0.001 Employer’s business closed temporarily due to COVID-19 (vs voluntarily not working) OR *p*-value 1.277 <0.001 Employer went out of business (vs voluntarily not working) OR *p*-value 1.617 <0.001
Zhou* et al * [81]	Laid off, furloughed, or otherwise unemployed due to COVID-19, unemployed prior to COVID-19	Laid off, furloughed, or otherwise unemployed due to COVID-19 = 103 (10.34%) at wave 1 45(7.15%) at wave 2 37(8.39%) at wave 3	DASS-21 with a cut-off 15	Moderate anxiety at wave 1: 10.38%, wave 2: 9.16%, wave 3: 9.05% Severe anxiety at wave 1: 8.03%; wave 2: 5.85%; wave 3: 3.39%. Extremely severe anxiety at wave 1: 26.34%; wave 2: 23.06%; wave 3: 21.27%	Sex, age, race, ethnicity, region, party identification, health condition, COVID-19 symptoms, COVID-19 testing	Laid off (vs unemployed prior to COVID-19) (wave 1) β (95%CI) 0.03 (−0.05,0.12) Laid off (vs unemployed prior to COVID-19) (wave 2) β (95%CI) 0.004 (−0.08,0.08) Laid off (vs unemployed prior to COVID-19) (wave 3) β (95%CI) 0.004 (−0.08,0.09)	

*GAD-7* Generalised Anxiety Disorder Assessment – 7 items, *GAD-2* Generalised Anxiety Disorder Assessment – 2 items, *DASS-21* Depression, Anxiety, Stress Scale – 21, *STAI* State-Trait Anxiety Inventory, *BAI* Beck Anxiety Inventory, *AOR* Adjusted Odds Ratio, *RR* Relative Risk, *WFH* Working From Home

The remaining cross-sectional studies used “no change in employment” (i.e. attending work as before) as reference category. These all found a positive significant association between job loss and anxiety. Participants recruited by Iowa University working pre-pandemic and who experienced job loss, reported worse anxiety scores than those whose job was unchanged [54]. Warren and colleagues [68] recruited a mixed sample of healthcare workers (40%), non-healthcare essential workers (30%) and general population (30%) and found that being unemployed because of COVID-19 (vs. working from normal location) was associated with higher anxiety and higher odds of clinically significant anxiety. In their study looking at the relationship between income reduction and mental health symptoms, Zhao et al. [72] reported that participants who became unemployed were 5 times more likely to report anxiety compared with participants whose income remained unchanged. A cross-sectional survey conducted in Philadelphia (USA) showed a significant association between job loss and anxiety only among men [39]. While a survey of the Indian adult population revealed that those who were temporarily unemployed since lockdown were twice as likely to score positively for anxiety, as opposed to people still employed [53]. Smith et al. compared the adjusted proportions of participants with anxiety among those no longer working as opposed to site-based workers. They found that in the overall sample, participants no longer employed reported similar adjusted proportions of anxiety compared with participants still working on site [65].

Three studies used people who were working from home during lockdown as a reference category. The first study recruited online just over 4000 residents in Kuwait and reported that no longer working or studying was associated with slightly increased odds of anxiety [38]. While a smaller study with data collected in multiple countries reported that losing job/business during lockdown was associated with higher anxiety score [55]. Finally, results from the Mental Health and Wellbeing Survey during COVID-19 Pandemic performed in the US found that no longer working due to COVID-19 was not associated with anxiety [51].

#### Working from home and anxiety

Three longitudinal studies explored the association between working from home and anxiety. The study by Batterham et al. [73] found that being able to work from home (vs not) was associated with a greater decline in anxiety over the course of the follow-up time (3 months). Among a cohort of Finnish workers [79], there was no increase in anxiety in those who became home workers since lockdown. However, the authors found that being a home worker in 2020 was cross-sectionally associated with an increase in anxiety. Finally, the study by Dragano et al. [76] reported that working from home in lockdown was associated with increased anxiety. The remaining studies exploring the effect of working from home on anxiety were cross-sectional. All studies except two found no significant association between working from home (either shift to home working or home working in lockdown) and anxiety [40, 50, 51, 68]. In the study conducted by Smith and colleagues [65] however, participants working from home reported significantly lower prevalence of anxiety compared with participants site-based. Prata Ribeiro and colleagues found that working from home in lockdown was beneficial to anxiety compared with working from the workplace [60].

### Key findings of the “vote-counting” method

Findings from the 39 analytical studies were summarised with the aid of an effect of direction plot and are presented in Table 4, arranged by study design and study quality. Of the 30 cross-sectional studies presented in the table, 27 explored the association between job loss and anxiety, with 25 of those reporting a negative association between the two (i.e. job loss harmful to anxiety). Only one study reported job loss to be beneficial for anxiety and one study reported inconsistent findings and was therefore excluded from the calculation of the sign test. The two-tailed sign test *p*-value is < 0.001, implying convincing evidence of a negative association between job loss and anxiety. Eight of the nine longitudinal studies explored job loss as the exposure, seven of which showed it to increase anxiety while one found the opposite. The two-tailed sign test *p*-value is 0.04, suggesting evidence of a negative association between job loss and anxiety also among the longitudinal studies.

**Table 4:** Effect direction plot of the 39 analytical studies

Author, year	Study design	Country	Job loss	Working from home	Furloughed	Any of job loss, reduction working hours	Decrease working hours	Study quality
Dawel [40], 2020	CS	Australia	▼	▼				++
Fisher [44], 2020	CS	Australia	▼					++
Hammarberg [48], 2020	CS	Australia	▼^2^					++
Smith [65], 2020	CS	Canada	▼	▲				++
Abrams [35], 2021	CS	USA	▼	▼	▼		▼	++
Burstyn [39], 2021	CS	USA	◄►^2^					++
Guerin [46], 2021	CS	USA	▼				▲	++
McDowell [54], 2021	CS	USA	▼					++
Okafor [58], 2021	CS	USA	▼					++
Prata Ribeiro [60], 2021	CS	Portugal		▲				++
Umucu [67], 2021	CS	USA	▼					++
Warren [68], 2021	CS	USA	▼^2^	▼^2^				++
Zhao [72], 2021	CS	Hong Kong	▼					++
Zhang [71], 2022	CS	27 European Countries		▼				++
Settels [62], 2023	CS	27 European Countries	▼					++
Shahaj [63], 2023	CS	27 European Countries	▼					++
Wright [69], 2021	CS	UK	▼					++
Burhamah [38], 2020	CS	Kuwait	▼					+
Jewell [51], 2020	CS	USA	▼	▲				+
Nelson [57], 2020	CS	USA, Canada, Europe	▼					+
Mojtahedi [55], 2021	CS	UK, Ireland, North America, India, Brazil	▼^2^		▲^2^			+
Ruengorn [61], 2021	CS	Thailand	▼					+
Alsaif [36], 2022	CS	Saudi Arabia	▼					+
de Miquel [41], 2022	CS	Spain	▲					+
Hagen [47], 2022	CS	Norway	▼					+
Shalaby [64], 2022	CS	Canada	▼					+
Hoffmann [50], 2023	CS	Poland		▲				+
Mani [53], 2023	CS	India	▼					+
Monnig [56], 2023	CS	USA	▼					+
Zamanzadeh [70], 2023	CS	China, Italy, Japan, South Korea, UK, USA	▼					+
Savolainen [79], 2021	L	Finland	▼	▼				+++
Dragano [76], 2022	L	Germany	▼	▼				+++
Blomqvist [74], 2023	L	Sweden	▼		▲			+++
Batterham [73], 2021	L	Australia	▼	▼				++
Blomqvist 2 [75], 2023	L	Sweden	▲		▲			++
Zhou [81] , 2020	L	USA	▼					++
Yao [80], 2021	L	USA	▼					+
Matsubayashi [78], 2022	L	Japan				▼		+
Hwang [77], 2023	L	South Korea	▼				▲	+

Study desing: CS: cross sectional; L: longitudinal

Effect direction: upward arrow ▲= positive health impact, downward arrow ▼= negative health impact, sideways arrow ◄►= no change/mixed effects/conflicting findings

Sample size: Final sample size Large arrow ▲ >300; medium arrow ▲ 50–300; small arrow ▲ <50

Study quality: +++ = high quality; ++ = medium quality; + = acceptable

Number of outcomes analysed is 1 unless indicated otherwise by the superscript number next to the effect direction

A total of eight cross-sectional studies reported on the association between working from home in lockdown and anxiety: half showed a negative association and half reported a positive association between working from home and anxiety. The two-tailed sign test was not significant (*p* = 0.39) meaning that there is insufficient evidence to support an association between working from home and anxiety in any direction. Three of the nine longitudinal studies explored the effect of working from home on anxiety and all showed working from home increased levels of anxiety.

Only two cross-sectional studies evaluated the effect of being furloughed on anxiety and found opposite results, while the two longitudinal studies showed that furlough might have reduced levels of anxiety. However, data were too sparse for this association to be statistically significant.

Finally, only three studies included in this review looked at enforced reduced working hours as the exposure. The two cross-sectional studies produced conflicting results while the longitudinal study found a decrease in working hours following lockdown reduced levels of anxiety. One study had a composite exposure defined as “job loss or reduction in working hours” and found this increased the odds of anxiety.

### Findings among middle-aged people

Of particular interest to our review was the group of middle-aged people and, although they are represented in each of the 48 studies included (according to the study protocol), only 17 studies reproduced associations separately for this age group. Some of these findings were shown in the published manuscripts while others were obtained by contacting the corresponding authors.

Hammarberg et al. [48] conducted a short online survey in Australia and found that women aged 50+ who lost a job because of COVID-19 restrictions were 50% more likely to report clinically significant symptoms of anxiety than those who did not. Estimates for men were not significant. The study by Abrams et al. [35] only included Americans aged 55+ but failed to find a significant association between job loss or being furloughed, or working from home and anxiety (vs job unchanged).

Settels, Shahaj and Zhang all conducted secondary analyses of the Survey of Health, Ageing and Retirement in Europe (SHARE) which includes adults aged 50+ from 27 European countries. Twenty one percent of the sample in Zhang’s study said that they had felt more anxious or nervous than the previous month; however, the authors did not find a significant association between working from home (vs working in the usual workplace) and anxiety [71]. Shahaj used the same outcome and reported that participants who lost their job were twice as likely to have experienced increased anxiety in the previous month [63]. Settels found that those who had lost their employment or their business because of the pandemic were 70% more likely to have felt nervous, anxious or on edge in the previous month, compared to those who did not [62]. Most studies were underpowered for this stratified analysis and did not find significant associations between exposures and anxiety among people aged 50+ [40, 41, 46, 74, 75, 79]. However, loss of employment was significantly associated with increased risk of anxiety among people age 50+ in a series of studies [56, 76, 78, 80], and the proportion of clinically significant anxiety was higher among people with job loss vs those without [52]. Finally, working from home was linked with higher levels of anxiety among people aged 50+ in the study by Dragano [76], while the association was not significant in a study of Finnish workers [79].

## Discussion

In this systematic review, we aimed to combine evidence on the association between changes in employment enforced by the COVID-19 pandemic and anxiety among adults, aged 18 and older, and with a particular focus on people aged over 50. We identified 48 studies which met the inclusion criteria, the majority of which adopted a cross-sectional design. We found strong evidence of enforced job loss due to COVID-19 increasing the levels of, or risk of, anxiety, from both cross-sectional and longitudinal studies. We were unable to find conclusive evidence about the effect of working from home or of being furloughed on anxiety as data were too sparse to draw meaningful conclusions. Only three [74, 76, 79] of the 48 studies included, all longitudinal in design, were rated of “high-quality”, which is at least partly due to the methodological challenges of incepting new research during a global pandemic.

This systematic review had a particular focus on older workers, aged 50 and older. Although all papers included this age group, only 17 provided estimates for this age group only. Five papers reported an increased likelihood of anxiety for people who lost their employment after the beginning of lockdown in this age group overall [56, 62, 76, 78, 80], one paper among older working women only [48], while one paper identified job loss as a risk factor for deterioration of anxiety within the previous 4 weeks [63]. Only one paper found a significant association between working remotely and increased anxiety among adults aged 50+ [76].

The age group 50+ is of great importance as, while governments are implementing policies to encourage them to remain in paid employment to older ages, unexpected disruption to their employment occurred since the pandemic may result in a permanent departure from paid work. Moreover, middle-aged people are likely to live with multimorbidities. Data from the UK show that nearly 50% of 50–64-year-olds live with one long-term condition, while 23% live with 3 or more [13]. Consequently, the rise in anxiety resulting from employment-related changes since lockdown will add to their already impaired health and compromise their chances to ever return successfully to paid employment.

This review shows evidence that the disruption of normal work functioning since the beginning of the pandemic and related lockdowns has had some impact on anxiety levels in the adult population, with at least similar effects among workers aged 50+. We cannot exclude that similar associations may have been identified in different periods, given the well-established negative association between unemployment and mental health [8–10]. Nonetheless, these effects hold significance given the number of people affected by changes in employment since lockdown.

Unfortunately, since changes in employment occurred in parallel with other sources of stress such as financial worries, worries about infection, and mandatory isolation, we are unable to disentangle the effect of employment changes from those of other stressors, in their impact on anxiety. However, we have no reason to believe that these stressors may confound the association of interest. For example, financial worries are likely a consequence of job loss, while worries about infection would apply equally to those experiencing a job change and those who did not—and, if anything, might affect more strongly the reference group. Finally, mandatory isolation could be considered as a stressor affecting both groups and could be seen as a consequence of a job change and not a confounder of the association.

It is unlikely that combining the estimates in a meta-analysis would contribute added value due to the diversity of ways in which exposures and outcomes were assessed. However, in order to have an impression of the magnitude of the associations, we have pooled evidence from eight studies, all exploring job loss (assessed in a variety of ways) in relation to a dichotomous anxiety outcome, among individuals aged 50+. We found an overall OR of 1.67 (95% CI 1.39 to 1.93) which suggests a significant and moderate increased risk of anxiety for middle-aged people who have lost their job since the pandemic. While the effect is moderate at the individual level, it carries important public health implications given the number of people affected by job losses since the pandemic.

Our findings on the relationship between working from home in lockdown and anxiety are inconclusive. Whether working from home during lockdown caused anxiety to decrease or increase remains uncertain. We speculate that this result is possibly due to methodological differences across papers, as individuals working from home have been compared to a diverse range of other groups. Additionally, there is evidence that it is not working from home per se which might act as a stressor, but it is more an abrupt shift to working from home which might be unfavourable to health. Unfortunately only a handful of papers were able to discern whether participants had shifted to working from home since the beginning of lockdown [40, 51, 54, 79], while the majority simply classified them as working from home during lockdown.

These findings might have important implications as, in the post pandemic era, work practices have shifted and working from home, or at least hybrid working, has become more common than before the pandemic. Data from the European Union show that working from home was not common before the pandemic, with less than one in twenty employees reporting working from home regularly in 2018 and less than one in ten doing so occasionally [5]. Before the pandemic, working from home was usually restricted to certain types of work, and mostly done on an occasional basis, in order to reduce commuting times and to improve work-life balance [84]. It is therefore to be expected that all that was known about working from home in a “normal” scenario, might no longer be valid when the choice element is removed. With COVID-19 and lockdown, there was an abrupt shift to working from home and this moved from being a voluntary choice of the employee to being something imposed to them. Forty eight percent of employees in the EU reported working from home at least some of the time in July 2020 [5]. Similarly, the percentage of UK employees exclusively working from home rose from approximately 3% in January/February 2020 to over 30% in March/April 2020, during the peak of the first national lockdown [85].

In the current post-pandemic era, where hybrid work is widely adopted, a key challenge for employers is to account for employee’s preferences regarding work arrangements. Providing flexibility in work location and scheduling, aligned with the individual’s preferences, seems essential to warrant a good work-retention and good employee performance. In some circumstances, it may be advisable to manage employees’ expectations regarding work arrangements. At the same time, employers must ensure accountability by maintaining oversight of work output and performance to sustain the effectiveness of home working [86]. Different sectors of the population may have different needs in relation to hybrid working, with women more likely to be exposed to multiple household roles, and people from a low socio-economic position more likely to have a home environment less suitable for home working. Additionally, because of the increased importance of technology while working remotely, older workers may be in a more disadvantaged position and may need additional support for a healthy home working [87].

It is of course important to acknowledge that many jobs cannot be performed from home and that during the pandemic, these were the roles fulfilled by “essential workers” who continued working throughout. This manuscript has not focused on this important group of workers.

Some considerations about the methodology of papers included in the review need to be made. The main exposure of interest was self-reported and assessed in a variety of ways. Some papers assessed job loss by asking participants whether they lost their job since the beginning of lockdown, while others used different definitions or combined categories of exposure, making comparisons across studies challenging. For example, some papers combined losing the job with being temporarily unemployed (i.e. being furloughed). We believe this might not be methodologically correct as the effect of job loss and the one of furlough on mental health might not go in the same direction [35, 74, 75].

Because of different definitions of the exposure, the prevalence of job loss varied widely across studies. In most studies, it was generally around 8 to 15%: it reached 41% when it combined personal and household job loss [56], and 39% when the authors combined having stopped working with having stopped studying [38]. In a descriptive study set in a deprived neighbourhood of Johannesburg, as high as 70% of their sample reported to have lost the job since the COVID-19 pandemic [45].

Papers featuring in this review mostly originate from high-income countries with diverse cultures, welfare benefit payment, and healthcare systems and that have adopted different strategies to avoid the spread of the disease. On the one hand, such factors might pose an obstacle to the generalisability of these findings. On the other hand, the negative effect of job loss on anxiety has been found consistently across countries.

Anxiety was also self-reported and assessed with a variety of screening tools, some validated and some bespoke. Most studies that used the GAD-7 tool adopted the recommended cut-off score 10; however, others used different cut-off points. Five papers used a shorter version of GAD-7, namely GAD-2. Possibly due to the choice of different cut-off scores, the estimated prevalence of anxiety varied widely even across studies using the same screening tool. Nevertheless, the use of different tools to measure anxiety does not appear to have introduced bias in the association between employment changes and anxiety in the current review. Levels of anxiety reported in these studies are perhaps higher than expected. This is not surprising as the tools used in all surveys are screening tools and not diagnostic ones, and a recent study looking at mental health and wellbeing of healthcare workers during the pandemic in the UK, showed that the prevalence of common mental health disorders was significantly higher when using a screening tool compared to estimates obtained when using a diagnostic tool [88].

A strength of this review is the adherence to PRISMA guidelines throughout which ensures reproducibility, transparency and rigour of the review process. In addition, two reviewers screened independently a portion of titles and abstracts as well as performed data extraction and quality assessment. This ensured the process was consistent and rigorous. A possible limitation of the current review is that we restricted our search to peer-reviewed evidence only and did not include grey literature, which may have reduced publication bias. We mitigated this to some extent by contacting corresponding authors to request additional analysis for the 50+ age group, potentially capturing relevant findings that may not have been fully reported. Furthermore, a funnel plot for the odds ratio of anxiety in relation to job loss among older worker did not show evidence of asymmetry that would suggest publication bias or small-study effect. This was further confirmed by Egger’s test (*p* = 0.84). The search was also limited to literature published in either English or Italian. Although we might have missed potentially relevant material by adopting these filters, this is unlikely as most high-quality research is published in English. Ideally, this review would have incorporated only studies with a longitudinal design. Unfortunately, this would have yielded insufficient numbers of studies to analyse. Therefore, findings from cross-sectional and longitudinal studies have been reported, clearly separated throughout. It is reassuring however that the different study designs showed broadly similar findings. Additionally, it would have been useful if we could have compared the findings from studies that explored our research questions at different stages of the pandemic. Unfortunately, almost all included studies too place in the early phases of lockdown.

To have a more comprehensive selection of studies, we contacted all corresponding authors to ask for any additional analyses they could provide based on the age category 50 and older. We were unable to conduct a meta-analysis due to heterogeneity across studies in the assessment tools used to measure exposure and outcome; however, we complemented a narrative review with a vote-counting technique which has a quantitative component. Additionally, the association between employment changes and anxiety was not the main focus of all papers featuring in the review: in some papers, this was a secondary analysis only. We are aware that anxiety is only one aspect of the mental health strain that can derive from the COVID-19 pandemic. Further research on how changes in employment affected depression and other mental health outcomes is therefore needed.

## Conclusions

To the best of our knowledge, this systematic review is the first one to examine the impact of changes in employment circumstances enforced by the COVID-19 pandemic on anxiety of the adult population and older workers. This review found convincing evidence of a negative association between job loss and anxiety, but the effect size did not appear strong enough to justify significant concern. Nevertheless, if the association between job loss and anxiety was to persist long term, older workers who lost their job since the pandemic may deserve additional support. Considering the inevitability of future epidemics [89], Governments should ensure that the mental health of the general population is adequately supported.

## Supplementary Information

Additional File 1: Systematic Review Protocol.

Additional File 2: PRISMA Checklist.

Additional File 3: Full search strategy.

Additional File 4: Detailed quality assessment for studies included.

Additional File 5: Quality assessment tool.

## Data Availability

All data generated or analysed during this study are provided in tables within the main text or supplementary material.
