# Supplementary material for: The impact of changes to work circumstances enforced by COVID-19 on anxiety: a systematic review
Source: Syst Rev. 2025 Oct 15;14:195. doi: 10.1186/s13643-025-02950-9 (PMC12522775; doi:10.1186/s13643-025-02950-9)
Supplement: Supplementary file 1 — Additional File 1: Systematic Review Protocol. [file 13643_2025_2950_MOESM1_ESM.docx]

**Section 1: ADMINISTRATIVE INFORMATION**

*Item 1: Identification.* The effect of changes in employment that occurred since the COVID-19 pandemic on anxiety of the adult population: protocol for a systematic review.

*Item 2: Registration.* In accordance with the guidelines, our systematic review protocol was registered with the International Prospective Register of Systematic Reviews (PROSPERO) on 15/06/2021 (registration number CRD42021260499)

*Item 3a: Contact information.*

Corresponding author: Stefania D’Angelo, MRC Lifecourse Epidemiology Centre, University of Southampton, Southampton, UK, [sd@mrc.soton.ac.uk](mailto:sd@mrc.soton.ac.uk).

Other authors: Georgia Ntani (gn@mrc.soton.ac.uk), Holly Syddall (hes@mrc.soton.ac.uk), Karen Walker-Bone ([kwb@mrc.soton.ac.uk](mailto:kwb@mrc.soton.ac.uk)), Ilse Bloom ([ib2@mrc.soton.ac.uk](mailto:ib2@mrc.soton.ac.uk))

*Item 3b: Contributions.* SD is the guarantor. SD, GN, HES, KWB, IB drafted the protocol. All authors contributed to the development of the selection criteria, the risk of bias assessment strategy and data extraction criteria. SD developed the search strategy. All authors read, provided feedback, and approved the final manuscript.

*Item 4: Amendments.* If we need to amend this protocol, we will give the date of each amendment, describe the change, and give the rationale in this section. Changes will not be incorporated into the protocol.

*Item 5a: Sources.* N/A

*Item 5b: Sponsor.* N/A

*Item 5c: Role of sponsor and/or funder.* N/A

**Section 2: INTRODUCTION**

*Item 6: Rationale.*

Changes of employment status (e.g. unemployment, relocation to home-working and furloughing) have been common since the start of the COVID-19 pandemic in 2020. As previous studies have shown that job loss can be harmful for mental and physical health, we anticipate that people that have lost their job during the pandemic will have experienced worsening in their mental health. The effect of remote working on mental health is unclear.

*Item 7: Objectives.* The aim of this systematic review is to evaluate how changes in employment status that occurred with the COVID-19 pandemic have impacted people’s anxiety. The proposed systematic review will answer the following question:

What have been the effects of changes in employment status that occurred due the COVID-19 pandemic on anxiety of working-age adults?

**Section 3: METHODS**

*Item 8: Eligibility criteria.* Studies will be selected according to the criteria outlined below.

*Study designs*

We will include cohort studies, as well as cross-sectional or case-control studies.

*Participants*

Inclusion

We will include studies examining the general working adult population (aged 18 or over) as long as the group aged 50+ is represented. We will only include papers that explore how employment changes that occurred since the COVID-19 pandemic affected people’s anxiety.

Exclusion

We will exclude papers whose sample does not include the age group 50+. We will exclude papers that do not describe changes in job circumstances or status that occurred since the COVID-19 pandemic as a predictor of anxiety. We will also exclude papers focussing on specific occupational groups or on people with specific health conditions as these will not be generalisable to the population. We will exclude papers from qualitative studies as well as evidence from systematic reviews and/or meta-analyses.

*Intervention*

Of interest are studies looking at changes of employment status after the start of the pandemic and subsequent impact on anxiety.

*Comparison*

People whose job has not changed or has changed but not due to the pandemic.

*Outcomes*

Anxiety measured with any validated or not validated tool.

*Setting*

Studies included will be those carried out in any country, not just the UK

*Language*

We will include articles reported in English or Italian.

*Item 9: Information sources.*

Literature search strategies will be developed using medical subject headings (MeSH). We will search MEDLINE (OVID interface), EMBASE (OVID interface), Web of Science and PsycINFO (EBSCO). The literature search will be limited to the English or Italian languages.
To ensure literature saturation, we will scan the reference lists of included studies or relevant reviews identified through the search. Conference abstracts, editorials, notes, and letters will be excluded.

*Item 10: Search strategy.*

All published quantitative studies will be searched. No study design limits will be imposed on the search. Evidence from qualitative studies will not be included.

The specific search strategies will be developed with the support of a Health Services librarian. A draft MEDLINE search strategy is included in Appendix 1. PROSPERO will be searched for ongoing or recently completed systematic reviews. As relevant studies are identified, reviewers will check for additional relevant cited and citing articles.
Publication dates of papers will range from 01/01/2020 to the date when the search is performed. We anticipate the initial search to be performed in May 2022 and will be updated towards the end of the review (July 2023).

*Item 11a: Data management.*

Literature search results will be uploaded to EndNote and duplicates will be removed.

*Item 11b: Selection process.*

All extracted papers will then be exported to the online free software Rayyan. The review authors will then independently screen the titles and abstracts yielded by the search against the inclusion criteria.
We will obtain full reports for all titles that appear to meet the inclusion criteria or where there is any uncertainty. We will then screen the full text reports and decide whether these meet the inclusion criteria. We will seek additional information from study authors where necessary, to resolve questions about eligibility.

*Item 11c: Data collection process*

A bespoke data extraction form has been developed for the research question between the reviewers (Appendix 2). This will be completed independently by SD and another member of the team. Any disagreements will be discussed and where resolution cannot be achieved, a third reviewer (KWB) will provide a final decision.

*Item 12: Data items*

- Author, Year, Study type (research article, report, conference paper), Country
- Study design
- Eligibility criteria met (yes/no/unclear)
- Age and gender of sample
- Study sample description: a description of the sample, recruitment technique and recruitment period
- Definition of main exposure/s used (job loss, unemployment, remote work etc.)
- Definition of the health outcome/s used: details of tools used to measure anxiety
- Number and percentages with the exposure/s of interest
- Number and percentages or summary statistics of the outcome/s of interest (depending of the nature of the outcome/s)
- Methodology used to control for confounders in the analysis (adjusting for confounders, stratification, matching etc.)
- List of confounders considered
- Statistical methods used
- Results of the analysis (risk estimates with 95%CI)

*Item 13: Outcomes and prioritisation*

Outcomes of interest will be: anxiety, assessed with any tool. No secondary outcomes

*Item 14: Risk of bias individual studies*

Risk of bias will be assessed using a form based on the Scottish Intercollegiate Guidelines Network (SIGN) checklists which will be compiled for individual studies. This will be modified and piloted so that it is suitable for assessment of the risk of bias from the papers returned by the search terms. Separate forms will be created for case control studies and cohort studies as necessary.

After piloting, the risk of bias for each included paper will be independently assessed by two reviewers (SD and KWB) and any disagreement will be discussed. Risk of bias will be discussed and reported in the review.

*Item 15: Data synthesis.*

A systematic narrative synthesis will be provided with information presented in the text and tables to summarise and explain the characteristics and findings of the included studies.

We will use a vote counting approach based on direction of effect method, which consists in categorising each study’s effect estimates according to the direction of the exposure effect on the outcome (positive, negative, inconsistent).

We will consider a meta-analysis if the extracted measures are suitably homogeneous.

If practicable we will conduct a sensitivity analysis looking at adults aged 50+.
