# Supplementary material for: The impact of changes to work circumstances enforced by COVID-19 on anxiety: a systematic review
Source: Syst Rev. 2025 Oct 15;14:195. doi: 10.1186/s13643-025-02950-9 (PMC12522775; doi:10.1186/s13643-025-02950-9)
Supplement: Supplementary file 3 — Additional File 3: Full search strategy. [file 13643_2025_2950_MOESM3_ESM.docx]

**S2: Full Search Strategy**

| **Database** | **Search Terms** |
| --- | --- |
| Medline (Ovid) | 1. Unemployment/  2. Employment/  3. ((work* or job*OR employ*) adj3 (loss or losing or exit or chang*)).mp.  4. "unemploy*".mp.  5. "employ*".mp.  6. "furlough*".mp.  7. 1 or 2 or 3 or 4 or 5 or 6  8. ((exp Coronavirus/ or Coronavirus Infections/ or pneumonia virus*.mp. or cov.mp.) and (outbreak or wuhan or novel or '19' or '2019' or epidem* or epidemy or epidemic* or pandem* or new).mp.) or (coronavirus* or 'corona virus*' or ncov or '2019ncov' or 'covid*' or "sars cov 2" or 'sars2' or sarscov2 or sarscov-2 or "ncov 2019" or "sars coronavirus 2" or "sars corona virus 2" or "severe acute respiratory syndrome cov 2" or "severe acute respiratory syndrome cov2" or "severe acute respiratory syndrome cov*").mp.  9. "lockdown*".mp.  10. "lock-down*".mp.  11. 8 or 9 or 10  12. Anxiety/  13. Anxiety Disorders/  14. anxi*.mp.  15. 12 or 13 or 14  16. 15 and 11 and 7  17. limit 16 to (yr="2020 -Current" and (english or italian)) |
| EMBASE Classic + Embase(Ovid) | 1. Unemployment/  2. Employment/  3. ((work* or job*OR employ*) adj3 (loss or losing or exit or chang*)).mp.  4. "unemploy*".mp.  5. "employ*".mp.  6. "furlough*".mp.  7. 1 or 2 or 3 or 4 or 5 or 6  8. exp severe acute respiratory syndrome coronavirus 2/ or coronavirus disease 2019/ or experimental coronavirus disease 2019/  9. (corona* adj1 (virus* or viral*)).mp.  10. (CoV not (Coefficien* or co-efficien* or covalent* or covington or covariant* or covarianc* or "cut-off value*" or "cutoff value*" or "cut-off volume*" or "cutoff volume*" or "combined optimi?ation value*" or "central vessel trunk" or CoVR or CoVS)).mp.  11. (coronavirus* or 2019nCoV* or 19nCoV* or "2019 novel*" or Ncov* or "n-cov" or "SARSCoV-2*" or "SARSCoV-2*" or SARSCoV2* or "SARS-CoV2*" or "severe acute respiratory syndrome*" or COVID*2).mp.  12. "lockdown*".mp.  13. "lock-down*".mp.  14. or/8-13  15. Anxiety/  16. Anxiety Disorder/  17. anxi*.mp.  18. 15 or 16 or 17  19. 18 and 14 and 7  20. limit 19 to (yr="2020 -Current" and (english or italian)) |
| PsycINFO (EBSCO) | 1. (DE "Anxiety" OR DE "Anxiety Disorders" ) OR ( anxi*)  2. DE "COVID-19" OR DE "Coronavirus" OR DE "Middle East Respiratory Syndrome" OR DE "Severe Acute Respiratory Syndrome" OR "corona virus" OR "sars-cov-2" OR "covid-19" OR "2019-ncov" OR "coronavirus" OR "covid 19" OR "lockdown*" OR "lock-down*"  3. DE "Unemployment" OR (work* OR job* OR employ*) N3 (loss OR losing OR exit OR chang*)) OR unemploy* OR DE "Job Loss" OR furlough* OR "employ*"  4. S1 AND S2 AND S3  **Limiters** - Published Date: 20200101-20230731; Language: English |
| CINAHL (EBSCO) | 1. (MH "Anxiety") OR "anxi*" OR (MH "Anxiety Disorders")  2. (MH "COVID-19") OR "covid-19" OR "covid19" OR "coronavirus" OR "corona virus" OR (MH "SARS-CoV-2") OR "lockdown*" OR "lock-down*" OR (MH "COVID-19 Pandemic") OR (MH "Coronavirus Infections+") OR (MH "Coronavirus+")  3. (MH "Unemployment") OR (work* OR job* OR employ*) N3 (loss OR losing OR exit OR chang*) OR (unemploy* OR furlough* ) OR employ*  4. S1 AND S2 AND S3  **Limiters** - Published Date: 20200101-20230731; Language: English, Italian |
