# Supplementary material for: The impact of changes to work circumstances enforced by COVID-19 on anxiety: a systematic review
Source: Syst Rev. 2025 Oct 15;14:195. doi: 10.1186/s13643-025-02950-9 (PMC12522775; doi:10.1186/s13643-025-02950-9)
Supplement: Supplementary file 4 — Additional File 4: Detailed quality assessment for studies included. [file 13643_2025_2950_MOESM4_ESM.docx]

**Agreed quality assessment for Systematic Review**

| **Paper** | **Clear question** | **Inclusion criteria** | **Description of subjects** | **Groups**  **similar** | **% dropout *** | **Comparison*** | **Outcome defined** | **Outcome measured** | **Exposure measured** | **Confounders**** | **CIs**** | **Clear association**** | **Generalisable** | **Quality** |
| --- | --- | --- | --- | --- | --- | --- | --- | --- | --- | --- | --- | --- | --- | --- |
| Abdalla | Y | Y | Y | Y | N/A | N/A | Y | Y | Y | N/A | N/A | N/A | Y | + |
| Abrams | Y | Y | Y | U | N/A | N/A | Y | Y | Y | Y | Y | N | Y | ++ |
| Alsaif | Y | N | Y | U | N/A | N/A | Y | Y | Y | Y | Y | Y | N | + |
| Amer | Y | Y | Y | U | N/A | N/A | Y | Y | Y | N/A | N/A | N/A | N | + |
| Batterham | Y | Y | Y | U | Y | N | Y | Y | Y | U | Y | N | Y | ++ |
| Blomqvist I | Y | Y | Y | Y | Y | N | Y | Y | Y | Y | Y | N | Y | +++ |
| Blomqvist 2 | Y | Y | Y | Y | N | N | Y | Y | Y | N | Y | N | Y | ++ |
| Burhamah | Y | Y | Y | U | N/A | N/A | Y | N | N | U | Y | N | U | + |
| Burstyn | Y | Y | Y | U | N/A | N/A | Y | Y | Y | Y | Y | N | Y | ++ |
| Dawel | Y | Y | Y | Y | N/A | N/A | Y | Y | Y | Y | N | N | Y | ++ |
| De Miquel | Y | Y | Y | Y | N/A | N/A | Y | Y | N | N | Y | N | U | + |
| Dragano | Y | Y | Y | Y | N | N | Y | Y | Y | N | Y | Y | Y | +++ |
| Elezi | Y | Y | Y | U | N/A | N/A | Y | Y | Y | N/A | N/A | N/A | N | + |
| Fiorenzato | Y | Y | Y | U | N/A | N/A | Y | Y | Y | N/A | N/A | N/A | Y | + |
| Fisher I | Y | Y | Y | U | N/A | N/A | Y | Y | Y | N | Y | Y | Y | ++ |
| Fisher 2 | Y | Y | Y | U | N/A | N/A | Y | U | Y | N/A | N/A | N/A | N | + |
| Guerin | Y | Y | N | Y | N/A | N/A | Y | Y | Y | N | Y | Y | Y | ++ |
| Hagen | Y | Y | Y | U | N/A | N/A | Y | Y | Y | U | N | N | N | + |
| Hammarberg | Y | Y | Y | U | N/A | N/A | Y | Y | Y | N | Y | Y | Y | ++ |
| Haynes | Y | N | Y | U | N/A | N/A | Y | U | Y | N/A | N/A | N/A | N | 0 |
| Hoffmann | Y | Y | Y | U | N/A | N/A | Y | Y | Y | N | Y | N | N | + |
| Hwang | Y | Y | Y | Y | N | N | Y | U | Y | N | Y | Y | N | + |
| Jewell | Y | Y | Y | U | N/A | N/A | Y | Y | Y | N | N | N | N | + |
| Killgore | Y | Y | Y | U | N/A | N/A | Y | Y | Y | N/A | N/A | N/A | Y | + |
| Mani | Y | Y | Y | U | N/A | N/A | Y | Y | Y | N | N | Y | N | + |
| Matsubayashi | Y | N | Y | Y | N | N | Y | Y | N | N | Y | Y | U | + |
| McDowell | Y | Y | N | U | N/A | N/A | Y | Y | Y | Y | Y | Y | N | ++ |
| Mojtahedi | Y | Y | Y | U | N/A | N/A | Y | Y | Y | N | N | U | U | + |
| Monnig | Y | Y | Y | U | N/A | N/A | Y | Y | N | N | Y | N | N | + |
| Nelson | Y | Y | Y | U | N/A | N/A | Y | Y | U | N | Y | N | N | + |
| Okafor | Y | Y | Y | U | N/A | N/A | Y | U | N | Y | Y | Y | Y | ++ |
| Pieh | Y | Y | N | Y | N/A | N/A | Y | Y | Y | N/A | N/A | N/A | Y | + |
| Prata Ribeiro | Y | Y | Y | U | N/A | N/A | Y | Y | Y | Y | Y | Y | N | ++ |
| Ruengorn | Y | Y | Y | U | N/A | N/A | Y | N | Y | Y | Y | U | N | + |
| Savolainen | Y | U | Y | Y | Y | N | Y | Y | Y | Y | Y | N | Y | +++ |
| Settels | Y | Y | Y | Y | N/A | N/A | Y | N | Y | Y | Y | Y | Y | ++ |
| Shahaj | Y | Y | Y | Y | N/A | N/A | Y | N | Y | Y | Y | Y | Y | ++ |
| Shalaby | Y | N | Y | U | N/A | N/A | Y | Y | Y | N | Y | N | U | + |
| Smith | Y | Y | Y | U | N/A | N/A | Y | Y | Y | Y | Y | Y | Y | ++ |
| Solomou | Y | Y | Y | U | N/A | N/A | Y | Y | Y | N/A | N/A | N/A | N | + |
| Umucu | Y | Y | Y | U | N/A | N/A | Y | Y | Y | Y | Y | Y | U | ++ |
| Warren | Y | Y | Y | Y | N/A | N/A | Y | Y | Y | Y | Y | Y | Y | ++ |
| Wright | Y | Y | Y | U | Y | N | Y | Y | Y | U | Y | N | Y | ++ |
| Yao | Y | N | Y | Y | N | N | Y | Y | Y | Y | N | Y | U | + |
| Zamanzadeh | Y | N | Y | U | N/A | N/A | Y | U | Y | N | Y | Y | Y | + |
| Zhang | Y | Y | Y | Y | N/A | N/A | Y | U | Y | Y | Y | N | Y | ++ |
| Zhao | Y | Y | Y | U | N/A | N/A | Y | Y | Y | Y | Y | Y | Y | ++ |
| Zhou | Y | U | Y | U | Y | Y | Y | Y | Y | Y | Y | N | Y | ++ |

* Only applicable to longitudinal studies

** Only applicable to analytical studies

Y = yes; N = no; U = unclear; N/A = not applicable.

Quality: 0=Unacceptable; +=Acceptable; ++=Medium Quality; +++=High quality
