# Supplementary material for: The impact of changes to work circumstances enforced by COVID-19 on anxiety: a systematic review
Source: Syst Rev. 2025 Oct 15;14:195. doi: 10.1186/s13643-025-02950-9 (PMC12522775; doi:10.1186/s13643-025-02950-9)
Supplement: Supplementary file 5 — Additional File 5: Quality assessment tool. [file 13643_2025_2950_MOESM5_ESM.docx]

The risk of bias tool was developed based on a combination of the Scottish Intercollegiate Guidelines Network (SIGN) template and the Joanna Briggs Institute (JBI) checklist. Wording of some questions was slightly adapted to fit well with this systematic review. Items with an asterisk (*) are only applicable to cohort studies and not to cross-sectional studies.

**Title:**

**First Author and ID number:**

**Assessor:**

|  | **Yes** | **No** | **Unclear** | **Not applicable** | **Comments** |
| --- | --- | --- | --- | --- | --- |
| 1. The study addresses an appropriate and clearly focused question |  |  |  |  |  |
| **PARTICIPANTS** |  |  |  |  |  |
| 2. Were the criteria for inclusion in the sample clearly defined? |  |  |  |  |  |
| 3. Were the study subjects and the setting described in detail? |  |  |  |  |  |
| 4. The two groups being studied (exposed/not exposed) are selected from source populations that are comparable in all respects other than the factor under investigation |  |  |  |  |  |
| 5. The percentage of participants who dropped out before the study was completed is given * |  |  |  |  |  |
| 6. Comparison is made between full participants and those lost to follow-up, by exposure status* |  |  |  |  |  |
| **ASSESSMENT** |  |  |  |  |  |
| 7. The outcome (anxiety) is clearly defined |  |  |  |  |  |
| 8. The outcome (anxiety) is measured in a valid and reliable way |  |  |  |  |  |
| 9. The exposure was measured in a valid and reliable way |  |  |  |  |  |
| **CONFOUNDING** |  |  |  |  |  |
| 10. The main potential confounding factors were identified and considered in the design and analysis ± |  |  |  |  |  |
| **STATISTICAL ANALYSIS** |  |  |  |  |  |
| 11. Confidence intervals have been provided ± |  |  |  |  |  |
| **OVERALL ASSESSEMENT** |  |  |  |  |  |
| 12. Taking into account clinical considerations, your evaluation of the methodology used, and the statistical power of the study, do you think there is clear evidence of an association between exposure and anxiety? ± |  |  |  |  |  |
| 13. Are the results of this study potentially generalizable to other older workers? |  |  |  |  |  |

* Only applicable to cohort studies

± Not applicable to descriptive studies

|  | High quality (+++) | Medium quality (++) | Acceptable (+) | Unacceptable (0) |
| --- | --- | --- | --- | --- |
| 14. How well was the study done to minimise risk of bias/confounding |  |  |  |  |
